# Integrated LC–MS/MS profiling, network pharmacology, molecular docking, and enzyme inhibition assays reveal the antidiabetic potential of *Mitragyna speciosa*

**DOI:** 10.1016/j.toxrep.2026.102271

**Published:** 2026-05-14

**Authors:** Entris Sutrisno, Khoirunnisa Muslimawati, Jajang Japar Sodik, Nabila Hadiah Akbar, Rifky Rahmadi Khaerulihsan, Ihsan Jaya Fathurohman, Taufik Muhammad Fakih

**Affiliations:** a Department of Pharmacology and Clinical Pharmacy, Faculty of Pharmacy, Universitas Bhakti Kencana, Jl. Soekarno Hatta, Bandung 40614, Indonesia; b Pharmacist Professional Education Study Program (PPSP), Faculty of Mathematics and Natural Sciences, Universitas Lambung Mangkurat, Jl. Brigjen H. Hasan Basri, Banjarmasin 70123, Indonesia; c Laboratorium Genomik, Laboratorium Terpadu, Universitas Lambung Mangkurat, Jl. Jend. A. Yani, Banjarmasin 70123, Indonesia; d Department of Pharmaceutical Analysis and Medicinal Chemistry, Faculty of Pharmacy, Universitas Bhakti Kencana, Jl. Soekarno Hatta, Bandung 40614, Indonesia; e Pharmacist Professional Education Study Program (PPSP), Faculty of Mathematics and Natural Sciences, Universitas Islam Bandung, Jl. Batik Halus, Bandung 40123, Indonesia; f Department of Pharmacy, Faculty of Mathematics and Natural Sciences, Universitas Islam Bandung, Jl. Ranggagading, Bandung 40116, Indonesia

**Keywords:** *Mitragyna speciosa*, Diabetes mellitus, LC–MS/MS metabolite profiling, Network pharmacology, In vitro studies

## Abstract

*Mitragyna speciosa* has gained attention as a medicinal plant with potential metabolic benefits, yet its antidiabetic mechanism remains unclear. This study characterized the metabolite profile of *Mitragyna speciosa* and explored its potential antidiabetic mechanisms using LC–MS/MS, network pharmacology, molecular docking, and in vitro enzyme inhibition assays. Metabolites were identified by LC–MS/MS, followed by target prediction and collection of diabetes-related targets to obtain overlapping genes. Protein–protein interaction (PPI) analysis and maximal clique centrality (MCC) were used to determine hub proteins. Molecular docking evaluated binding affinity (ΔG) and inhibition constant (Ki) against prioritized targets, and docking accuracy was confirmed by re-docking RMSD. The predicted antidiabetic activity was subsequently validated through in vitro α-glucosidase and α-amylase inhibitory assays. LC–MS/MS revealed mitrafoline (31.54%) and mitragynine (30.59%) as dominant constituents, followed by corynoxine (10.43%), methyl indoloquinolizine derivatives (8.41%), and isopaynantheine (6.27%). Minor metabolites included mitraphylline (2.58%) and corynantheidine (1.75%). Network pharmacology identified 28 shared targets between *Mitragyna speciosa* and diabetes mellitus, forming an interconnected PPI network. Hub analysis prioritized EGFR (915), PIK3CA (808), JAK2 (673), SRC (606), and ERBB2 (602). Docking showed that corynantheidine had the strongest affinity for EGFR (ΔG −9.01 kcal/mol; Ki 250.21 nM), while mitraphylline performed best against JAK2 (ΔG −8.77 kcal/mol; Ki 374.56 nM); native ligands remained superior for PIK3CA, SRC, and ERBB2. In vitro assays revealed moderate α-glucosidase inhibitory activity of the extract (IC₅₀ ≈ 48.49 ppm) and substantially weaker α-amylase inhibition (IC₅₀ ≈ 17,907.37 ppm), indicating a selective enzyme inhibition profile. These findings suggest that *Mitragyna speciosa* exerts antidiabetic effects through multitarget modulation of kinase-centered signaling pathways and selective inhibition of carbohydrate-digesting enzymes.

## 1 Introduction

Diabetes mellitus (DM) is a progressive metabolic disorder marked by chronic hyperglycemia caused by impaired insulin secretion, reduced insulin sensitivity, or a combination of both [1]. Its global prevalence continues to rise and has become a major public health concern due to its long-term complications affecting the cardiovascular system, kidneys, nerves, and other tissues [10]. Recent epidemiological reports estimate that more than 500 million adults worldwide are currently living with diabetes, and this number is projected to increase substantially in the coming decades, emphasizing the urgency of improved prevention and therapeutic strategies [42]. Type 2 diabetes mellitus (T2DM) accounts for the majority of cases and is closely linked to obesity, sedentary lifestyle, and metabolic syndrome. Beyond abnormal glucose regulation, T2DM involves dyslipidemia, oxidative stress, and persistent inflammatory responses that worsen insulin resistance over time [2]. These interconnected disturbances indicate that diabetes cannot be fully explained by a single defective pathway, but rather by a complex network of molecular events. Current pharmacological treatments remain essential, yet limitations such as adverse effects, variable patient response, and the need for long-term therapy encourage continued exploration of alternative strategies [41]. Plant-derived metabolites are increasingly investigated because they offer chemically diverse scaffolds capable of interacting with multiple biological targets. This multitarget potential is particularly valuable for chronic diseases with complex pathogenesis such as T2DM [15]. Therefore, systematic evaluation of medicinal plants remains a relevant direction for discovering novel antidiabetic candidates.

*Mitragyna speciosa* (kratom) is a tropical plant traditionally used in Southeast Asia and has attracted increasing attention due to its broad pharmacological profile. Kratom is widely recognized for its alkaloid-rich composition, particularly indole and oxindole derivatives, which are associated with diverse biological activities [14], [6]. The complexity of kratom chemistry suggests that its bioactivity is unlikely to be driven by a single metabolite, but rather by the combined contribution of multiple compounds. This feature is relevant for diabetes research because multitarget modulation may address different pathological components simultaneously, including glucose dysregulation and metabolic stress [29], [54]. However, despite its popularity and expanding scientific interest, the antidiabetic mechanism of *Mitragyna speciosa* remains insufficiently clarified at the molecular level. Many studies report biological effects without systematically linking identified metabolites to disease-relevant targets and pathways [33], [38]. Establishing such mechanistic links is essential for strengthening scientific credibility and supporting future pharmacological development. In addition, chemical composition can vary depending on extraction conditions and sample origin, making metabolite-based confirmation an important step in research standardization. For this reason, analytical profiling techniques such as liquid chromatography–tandem mass spectrometry (LC–MS/MS) are crucial for mapping the chemical landscape of kratom extracts. LC–MS/MS data can provide a rational foundation for selecting representative compounds for further mechanistic evaluation.

Modern drug discovery increasingly recognizes that complex diseases such as T2DM involve dynamic interactions among signaling pathways rather than isolated enzymatic processes. Molecular networks regulating insulin signaling, glucose uptake, lipid metabolism, inflammation, and oxidative stress are interconnected and can compensate for single-target inhibition [34], [45]. Hence, multitarget strategies may provide more stable therapeutic outcomes by modulating several control nodes simultaneously. Network pharmacology has emerged as a useful framework for investigating herbal medicines because it can map relationships between plant metabolites, predicted protein targets, and disease-associated genes. This approach enables identification of shared targets and prioritization of hub proteins that hold high regulatory influence in protein–protein interaction networks [31]. Hub targets are particularly valuable because they often represent upstream regulators that affect multiple downstream biological processes relevant to diabetes progression. In addition, enrichment analyses such as GO and KEGG can highlight biological themes and pathways that are overrepresented among the shared targets, offering functional interpretation [47]. However, network pharmacology alone cannot confirm whether metabolites can physically interact with the predicted proteins. Therefore, molecular docking is commonly applied to validate binding feasibility by predicting binding affinity, inhibition constants, and residue-level interactions within target active sites. The integration of LC–MS/MS, network pharmacology, and docking thus provides a robust framework for mechanistic exploration of plant-based antidiabetic candidates.

A critical step in such an integrated workflow is the selection of representative compounds and biologically meaningful targets for docking validation. Compound selection should ideally consider both chemical evidence from LC–MS/MS and mechanistic relevance based on network centrality. Similarly, target selection should prioritize proteins that are strongly associated with diabetes pathophysiology and appear as hub regulators within the interaction network [36], [50]. Structural docking requires reliable receptor structures and careful parameterization to ensure that predicted poses are meaningful and reproducible. Re-docking validation is also important to confirm that the docking protocol can reproduce the binding orientation of native ligands within acceptable RMSD thresholds [24]. Once validated, docking outputs can provide quantitative evidence supporting which metabolites are most likely to engage hub proteins effectively. Residue interaction analysis further strengthens interpretation by identifying conserved binding pocket residues and potential stabilizing interactions. Such mechanistic insights are essential for selecting candidate compounds for downstream experimental testing. In diabetes research, this type of prioritization can guide targeted validation such as enzyme inhibition assays, glucose uptake studies, and biomarker evaluation [22], [35]. In particular, in vitro assays targeting carbohydrate-digesting enzymes such as α-glucosidase and α-amylase are widely used to assess the ability of plant extracts to regulate postprandial glucose levels, providing functional validation of predicted antidiabetic activity. Therefore, a structured computational pipeline can bridge chemical identification and biological relevance in a scientifically defensible manner.

Accordingly, this study aimed to characterize the metabolite profile of *Mitragyna speciosa* using LC–MS/MS and to elucidate its antidiabetic mechanism through an integrated network pharmacology and molecular docking strategy. LC–MS/MS analysis was performed to identify major and minor metabolites and to establish a chemical basis for compound prioritization [48]. Network pharmacology was applied to predict potential targets of the identified compounds and to determine overlapping genes associated with diabetes mellitus. Protein–protein interaction analysis was then conducted to identify hub proteins that may serve as key regulatory nodes in the antidiabetic mechanism of kratom [15]. Molecular docking was subsequently performed to evaluate the binding affinity and predicted inhibition constants of selected compounds against prioritized hub targets. Re-docking validation was included to ensure reliability and reproducibility of docking parameters. Residue-level interaction profiling was used to support mechanistic interpretation of ligand–protein binding patterns [9]. In addition, α-glucosidase and α-amylase inhibition assays were conducted to experimentally validate the predicted antidiabetic potential at the enzyme level. Through this approach, the study provides systematic insight into how kratom metabolites may modulate diabetes-associated molecular pathways. The findings are expected to support rational compound selection for further in vitro and in vivo validation. Ultimately, this integrated framework contributes to strengthening the evidence base for *Mitragyna speciosa* as a potential multitarget antidiabetic candidate.

## 2 Materials and Methods

### 2.1 Materials and Reagents

All solvents and reagents used in this study were of analytical grade or higher to ensure reliable chemical profiling and downstream computational interpretation. Methanol (LC–MS/MS grade) and acetonitrile (LC–MS/MS grade) were purchased from Merck (Darmstadt, Germany) and used as extraction solvents and mobile phase components for chromatographic separation. Formic acid (≥98%, LC–MS/MS grade) was also obtained from Merck (Darmstadt, Germany) and applied as a mobile phase modifier to enhance electrospray ionization efficiency and improve peak shape. Ultrapure water was produced in-house using a Milli-Q water purification system (Millipore, MA, USA) and used for preparation of aqueous mobile phases. Nylon syringe filters (0.22 µm) and LC–MS/MS compatible sample vials were purchased from standard laboratory suppliers and were used to remove particulates and prevent injector clogging. All solvents were freshly prepared prior to analysis, and mobile phases were degassed to minimize baseline noise and signal fluctuation. Plant sample preparation was conducted under clean laboratory conditions to reduce cross-contamination across sample batches. LC–MS/MS instrument performance was monitored using routine system suitability checks, including stable retention time and consistent mass accuracy. Metabolite annotation was carried out using high-resolution mass spectrometry data and comparison with public spectral databases, enabling putative identification without external reference standards. All analytical steps were performed following standard laboratory procedures to ensure reproducibility and traceability.

### 2.2 Plant Material and Extract Preparation

Leaves of *Mitragyna speciosa* were used as the study sample and processed prior to metabolite profiling. The collected leaves were washed briefly with running water to remove dust and surface contaminants, followed by rinsing with distilled water. The plant material was then dried in a ventilated drying oven at 40 °C for 48–72 h until a constant weight was achieved to minimize moisture-related degradation. Dried leaves were pulverized using a laboratory grinder and sieved to obtain a fine and homogeneous powder. For extraction, 100 g of leaf powder was mixed with 1,000 ml of 70% ethanol (v/v) in a closed glass container. The mixture was macerated at room temperature (25 ± 2 °C) for 72 h, with gentle agitation performed for 10 min every 8 h to improve solvent penetration and metabolite diffusion. After the first maceration cycle, the mixture was filtered through Whatman No. 1 filter paper, and the plant residue was re-extracted twice under identical conditions to maximize compound recovery. The combined filtrates were pooled and concentrated under reduced pressure using a rotary evaporator at 40 °C to remove ethanol and obtain a viscous crude extract. The concentrated extract was then dried using a freeze-dryer (lyophilizer) for 24–48 h to yield a stable dry extract suitable for LC–MS/MS analysis. Prior to injection, 10 mg of dried extract was reconstituted in 1 ml methanol (LC–MS/MS grade) and vortex-mixed for 2 min. The solution was sonicated for 10 min to ensure complete dissolution and then centrifuged at 10,000 rpm for 10 min to remove insoluble particles. The supernatant was passed through a 0.22 µm nylon syringe filter and transferred into LC–MS/MS vials. Prepared samples were stored at 4 °C and analyzed within 24 h to maintain metabolite stability and prevent chemical degradation [52].

### 2.3 LC-MS/MS Analysis and Metabolite Annotation

Metabolite profiling of *Mitragyna speciosa* leaf extract was conducted using an ultra-high-performance liquid chromatography (UHPLC) system coupled with a high-resolution tandem mass spectrometer (HRMS/MS) equipped with an electrospray ionization (ESI) source. The analytical workflow comprised sequential stages including sample preparation, chromatographic separation, high-resolution mass spectrometric acquisition, and multi-criteria metabolite annotation. Prior to analysis, the dried ethanolic extract was reconstituted in LC–MS/MS grade methanol, vortex-mixed, and filtered through a 0.22 µm PTFE membrane to remove particulate matter and ensure column protection. Chromatographic separation was performed on a reversed-phase C18 column (100 mm × 2.1 mm, 1.8 µm particle size) maintained at 35 °C to ensure retention time stability and optimal peak resolution. The mobile phase consisted of solvent A (0.1% formic acid in ultrapure water, v/v) and solvent B (0.1% formic acid in acetonitrile, v/v), selected to promote protonation and enhance ionization efficiency of alkaloid-dominant metabolites. A gradient elution program was applied as follows: 0–2 min, 5% B; 2–12 min, 5–40% B; 12–18 min, 40–95% B; 18–20 min, 95% B; followed by re-equilibration to 5% B from 20 to 22 min. The flow rate was maintained at 0.30 ml/min, with an injection volume of 2 µL per run, while the autosampler temperature was set at 4 °C to minimize analyte degradation during batch analysis. Mass spectrometric detection was carried out in positive ion mode (ESI+), which is particularly suitable for the detection of protonated indole and oxindole alkaloids characteristic of *Mitragyna speciosa*. The ESI source parameters were optimized as follows: drying gas temperature 325 °C, drying gas flow 10 L/min, nebulizer pressure 35 psi, sheath gas temperature 350 °C, and sheath gas flow 11 L/min. The capillary voltage was set at 3500 V, while the fragmentor voltage was maintained at 150 V to ensure efficient ion transmission. Full-scan mass spectra were acquired over an *m/z* range of 100–1000, enabling comprehensive coverage of semi-polar and moderately lipophilic metabolite classes. Data-dependent MS/MS acquisition was employed to support structural elucidation, in which precursor ions detected in full-scan mode were automatically selected for fragmentation using stepped collision energies ranging from 20 to 40 eV. This approach generated diagnostic product ion patterns essential for confirming core structural features of candidate metabolites. Continuous internal mass calibration was applied using reference ions at *m/z* 121.0509 and 922.0098, ensuring high mass accuracy throughout the analytical sequence. Raw chromatographic and spectral data were processed using the instrument’s proprietary software, including peak detection, deconvolution, and extraction of ion chromatograms (EICs). Putative metabolite identification was achieved through a multi-parameter annotation strategy, integrating accurate mass measurement (ppm error), isotopic distribution, and MS/MS fragmentation behavior. Spectral features were matched against established public databases, including METLIN, MassBank, and PubChem. Relative metabolite abundance was estimated based on peak area percentages derived from extracted ion chromatograms, providing a semi-quantitative comparison of compound distribution within the extract. All identified metabolites were classified as tentative due to the absence of authentic reference standards, and the results were therefore interpreted within a qualitative-to-semiquantitative metabolomic framework [48].

### 2.4 Collection of Bioactive Compounds and Target Prediction

Metabolites annotated from LC–MS/MS profiling were used as the initial compound pool for network pharmacology analysis. Each compound was verified by retrieving its PubChem CID, 2D structure, and canonical SMILES from the PubChem database (https://pubchem.ncbi.nlm.nih.gov/) to ensure standardized molecular representation [25]. Physicochemical and pharmacokinetic screening was conducted using SwissADME (https://www.swissadme.ch/) to prioritize metabolites with favorable drug-like characteristics [13]. The screening parameters included molecular weight, lipophilicity, topological polar surface area, hydrogen bond donors/acceptors, and rotatable bonds. Compounds predicted to have high gastrointestinal absorption were preferentially retained for downstream analysis. Drug-likeness filters were applied, and compounds were selected if they met at least two criteria among Lipinski, Ghose, Veber, Egan, and Muegge rules. Metabolites that failed to satisfy the selection criteria were excluded to reduce low-confidence candidates. Target prediction for the retained compounds was performed using SwissTargetPrediction (https://www.swisstargetprediction.ch/) with the species restricted to Homo sapiens [17]. Predicted targets were collected based on the platform probability ranking and compiled for each compound. To expand the predicted target space, additional target prediction was conducted using the Similarity Ensemble Approach (SEA) server (https://sea.bkslab.org/) [56]. Targets obtained from both platforms were merged into a single dataset and duplicate entries were removed. All target identifiers were standardized by converting protein names into official gene symbols using the UniProt database (https://www.uniprot.org/) [5]. Targets that could not be mapped to valid human gene symbols were excluded from further analysis. The curated target list was subsequently used for overlap analysis with diabetes mellitus–associated targets. This workflow ensured that compound prioritization was grounded in LC–MS/MS evidence while target prediction remained systematic and reproducible.

### 2.5 In Silico Toxicity Prediction

To assess the preliminary safety profile of selected *Mitragyna speciosa* metabolites, in silico toxicity prediction was performed using the ProTox-3 web server (https://tox.charite.de/protox3/index.php?site=compound_input) [3]. Canonical SMILES strings of each compound were retrieved from the PubChem database and used as standardized molecular inputs for toxicity evaluation. Each SMILES structure was submitted individually through the ProTox-3 compound input interface to generate toxicity predictions. The platform was used to estimate the median lethal dose (LD₅₀, mg/kg) and to assign compounds into toxicity classes (Class I–VI) based on the predicted acute toxicity level. In addition, organ toxicity endpoints were assessed, including predicted hepatotoxicity, to provide early insight into potential safety risks. The toxicity-related endpoints evaluated also included carcinogenicity, mutagenicity, cytotoxicity, and immunotoxicity, which are commonly used to flag compounds with potential adverse biological effects. Prediction outputs were recorded for each metabolite and compiled into a comparative dataset to support compound ranking. Compounds classified into higher-risk toxicity groups (Class I–III) or showing positive toxicity alerts were considered less favorable for further development. The toxicity predictions were interpreted as preliminary computational estimates rather than definitive experimental conclusions [30]. ProTox-3 results were integrated with network pharmacology and docking outcomes to support balanced prioritization between predicted efficacy and safety. This approach enabled selection of candidate metabolites with both mechanistic relevance and acceptable predicted toxicity profiles for further validation.

### 2.6 Identification of Type 2 Diabetes Mellitus–Related Targets

Targets associated with type 2 diabetes mellitus (T2DM) were systematically collected by searching the keyword “type 2 diabetes mellitus” in multiple publicly accessible biomedical databases. The databases used in this study included PharmGKB (https://www.pharmgkb.org/) [4], DrugBank (https://go.drugbank.com/) [53], OMIM (https://www.omim.org/) [18], and GeneCards (https://www.genecards.org/) to ensure comprehensive coverage of genes and proteins linked to diabetes pathogenesis [43]. From each database, target entries were downloaded and compiled in spreadsheet format for further curation. Only targets annotated as Homo sapiens and categorized as protein-coding genes were retained to maintain clinical and biological relevance. For GeneCards, genes were filtered by selecting entries with relevance scores above the median value, thereby minimizing weakly associated targets and reducing background noise. Targets obtained from PharmGKB, DrugBank, and OMIM were collected based on their reported association with T2DM-related phenotypes, pathways, or therapeutic relevance. All targets from the four databases were merged into a unified dataset, and gene identifiers were standardized to official gene symbols to ensure consistency across platforms. Standardization was performed by cross-checking gene names using UniProt and eliminating ambiguous or outdated identifiers. Duplicate targets arising from overlapping database coverage were removed to obtain a final non-redundant list of T2DM-associated genes. The curated diabetes target set was subsequently used for intersection analysis with predicted compound targets derived from *Mitragyna speciosa* metabolites. This approach enabled identification of shared targets that may represent key molecular intervention points underlying the antidiabetic potential of kratom. All data handling steps were performed using a consistent workflow to improve reproducibility and transparency of target collection.

### 2.7 Network Construction and Hub Target Screening

An overlap analysis was performed to identify common genes between the predicted targets of *Mitragyna speciosa* metabolites and the curated T2DM-associated target set. The two target lists were first standardized to official gene symbols and then compared to generate a shared target dataset representing potential therapeutic intervention points. The overlap results were visualized using a Venn diagram generated through an online bioinformatics platform to provide a clear representation of intersecting genes. The overlapping genes were subsequently uploaded to the STRING database (http://string-db.org/) to construct a protein–protein interaction (PPI) network [49]. During STRING analysis, the organism was restricted to Homo sapiens, and only experimentally supported and curated interaction evidence channels were considered to improve biological reliability. A high-confidence interaction score threshold (≥0.70) was applied to reduce false-positive associations and retain robust functional relationships. The resulting PPI network was exported from STRING in a compatible format and imported into Cytoscape for further visualization and network topology analysis [46]. Network refinement was performed by removing isolated nodes when necessary to focus on the connected core network structure. Topological properties were calculated using Cytoscape network analysis tools, including degree centrality, betweenness centrality, and closeness centrality, to quantify node influence and connectivity. Degree centrality was used to represent the number of direct interactions per node, while betweenness centrality reflected the role of a node in controlling information flow across the network. Closeness centrality was used to estimate how efficiently a node could interact with other proteins within the network. To strengthen hub identification, maximal clique centrality (MCC) scoring was performed using the CytoHubba plugin, which prioritizes nodes that participate in highly connected subnetwork clusters [12]. Hub proteins were ranked according to MCC values, and the top-scoring targets were selected as key regulators potentially involved in the antidiabetic mechanism of *Mitragyna speciosa.* The highest-ranked hub proteins were subsequently used as candidate receptors for molecular docking to validate ligand–target interactions at the structural level. This network-driven screening strategy ensured that docking targets were selected based on both disease relevance and strong regulatory importance within the diabetes-related interaction network.

### 2.8 GO Functional Annotation and KEGG Pathway Enrichment

To elucidate the biological significance of the overlapping targets, Gene Ontology (GO) functional annotation and Kyoto Encyclopedia of Genes and Genomes (KEGG) pathway enrichment analyses were conducted [21], [23]. The list of intersecting genes obtained from the compound–disease overlap was used as the input dataset for enrichment analysis. The background gene universe was defined as all annotated Homo sapiens protein-coding genes to ensure statistical consistency. Functional enrichment was performed using a web-based annotation platform, and gene identifiers were uploaded in the form of official gene symbols with the organism restricted to Homo sapiens. GO enrichment analysis was carried out to classify the targets into three major categories, including biological process (BP), molecular function (MF), and cellular component (CC), allowing systematic interpretation of gene function. KEGG pathway analysis was performed to identify signaling pathways potentially involved in diabetes-related mechanisms such as glucose metabolism, insulin-associated signaling, inflammatory regulation, and cellular stress response. Statistical significance was evaluated using a hypergeometric test followed by multiple testing correction with the Benjamini–Hochberg false discovery rate (FDR), and adjusted p-values (q-values) < 0.05 were considered significant. To improve robustness, enriched terms were ranked based on adjusted p-values and gene ratios, highlighting biological themes with the strongest enrichment signals. Redundant terms with highly overlapping gene sets were filtered using similarity-based clustering to retain representative terms with the lowest adjusted p-values, improving clarity of interpretation. It should be noted that enrichment analysis was conducted using unweighted overlap targets without incorporation of expression or quantitative activity data; therefore, results were interpreted cautiously as hypothesis-generating. The enrichment outputs were exported and organized into structured tables containing term names, gene counts, enrichment ratios, and adjusted significance values. Visualization of enrichment results was performed using standard plotting approaches to generate bar plots and bubble plots, enabling intuitive comparison of enriched terms and pathways. Bubble plots were used to display enrichment significance and gene ratio simultaneously, while bar plots emphasized the most dominant functional categories. The enrichment results were subsequently integrated with PPI hub analysis to support biological interpretation of prioritized targets. These analyses provided mechanistic context for molecular docking by linking predicted hub proteins to relevant biological processes and signaling pathways. GO and KEGG enrichment strengthened the functional relevance of the compound–target–disease network and supported hypothesis generation regarding the antidiabetic mechanisms of *Mitragyna speciosa*.

### 2.9 Molecular Docking and Interaction Analysis

Molecular docking was conducted to assess the binding potential of selected *Mitragyna speciosa* metabolites toward the prioritized hub target proteins identified from network analysis. Ligand structures were obtained from the PubChem database and prepared as three-dimensional conformations using molecular modeling software prior to docking. Geometry optimization was applied to each ligand to obtain energetically stable conformers and minimize unfavorable steric strain. Protein crystal structures of the selected hub targets were downloaded from the RCSB Protein Data Bank (PDB) and screened for appropriate experimental quality before use [7]. Protein preparation was performed by removing crystallographic water molecules and non-essential co-crystallized components while maintaining residues relevant to the binding pocket architecture. Receptor structures were then processed using AutoDock Tools 4.2.3, including addition of polar hydrogen atoms and assignment of Gasteiger charges [16]. Ligands were also prepared in AutoDock Tools by defining rotatable bonds and converting structures into the PDBQT format required for docking. Docking calculations were performed by defining a grid box centered on the binding site of the native ligand to ensure adequate coverage of the active pocket region. The docking workflow was implemented using MGLTools 1.5.7 as the supporting platform for input preparation and configuration. Binding affinity scores were obtained for each ligand–target complex, and the best-ranked pose was selected based on the lowest predicted binding energy and plausible binding orientation. The selected docking conformations were subsequently analyzed to identify key ligand–protein interactions, including hydrogen bonding, hydrophobic contacts, π–π stacking, and π–alkyl interactions. Interaction visualization and 2D/3D mapping were carried out using BIOVIA Discovery Studio Visualizer to support structural interpretation of the docking results [8]. The docking outcomes were then integrated with the network pharmacology findings to provide mechanistic insight into how *Mitragyna speciosa* metabolites may modulate diabetes-associated hub proteins.

### 2.10 Alpha-Glucosidase Inhibitory Activity

The α-glucosidase inhibitory activity of the tested compounds was evaluated using a modified standard enzymatic method in a 96-well microplate format. This assay was designed to determine the ability of the compounds to inhibit α-glucosidase-mediated hydrolysis of a chromogenic substrate. Each reaction well contained 50 µL of phosphate buffer solution at a concentration of 100 mM and pH 6.8. A volume of 10 µL of α-glucosidase solution with an activity of 1 U/ml was then added into each well. Thereafter, 20 µL of either the test sample or the reference standard acarbose at various concentrations was introduced into the reaction mixture. The prepared mixture was pre-incubated at 37 °C for 15 min to allow sufficient interaction between the enzyme and the inhibitor. After this initial incubation period, 20 µL of 5 mM 4-nitrophenyl β-D-glucopyranoside was added as the substrate to initiate the enzymatic reaction. The reaction mixture was then incubated again at 37 °C for 20 min under controlled conditions. To terminate the enzymatic reaction, 50 µL of 0.1 M sodium carbonate was added to each well. The release of p-nitrophenol produced during substrate hydrolysis was used as an indicator of α-glucosidase activity. Absorbance was measured at 405 nm using a Multiskan multiplate reader (Thermo Scientific). A control reaction was prepared using phosphate buffer instead of the test sample while maintaining all other assay conditions identical. All experiments were carried out in triplicate and repeated in three independent experimental runs to ensure data reliability and reproducibility. The inhibitory effect of each sample was expressed as percentage inhibition based on the decrease in absorbance relative to the control group. The percentage of α-glucosidase inhibition was calculated using the equation Inhibition (%) = (1 − A/B) × 100, where A represents the absorbance in the presence of the test sample and B represents the absorbance of the control reaction [27].

### 2.11 Alpha-Amylase Inhibitory Activity

The α-amylase inhibitory activity of compounds 1–5 was determined using a modified standard enzymatic assay in a 96-well microplate. This method was applied to evaluate the capacity of the tested compounds to suppress starch hydrolysis catalyzed by α-amylase. Each reaction mixture consisted of 50 µL of phosphate buffer at a concentration of 100 mM and pH 6.8. A volume of 10 µL of α-amylase solution with an activity of 2 U/ml was added to each well. After enzyme addition, 20 µL of either the test sample or the standard solution at concentrations ranging from 31.2 to 100 µg/ml was introduced into the mixture. The reaction system was then incubated at 37 °C for 20 min to allow preliminary interaction between the enzyme and the tested inhibitor. Following this pre-incubation step, 10 µL of 1% soluble starch solution was added as the substrate. The resulting mixture was further incubated at 37 °C for 30 min to permit the enzymatic hydrolysis reaction to proceed. After completion of the reaction, 100 µL of DNS color reagent was added to each well. The microplate was then heated at 95 °C for 15 min to develop the color produced by reducing sugar formation. After the heating step, the reaction mixture was allowed to cool to room temperature before absorbance measurement. The absorbance was recorded at 450 nm using a multiplate reader to quantify the extent of enzymatic activity. A control group was prepared using phosphate buffer in place of the sample under the same experimental conditions. All measurements were performed in triplicate and repeated in three independent experiments to ensure consistency of the obtained results. The percentage of α-amylase inhibition was calculated using the equation Inhibition (%) = (1 − A/B) × 100, where A represents the absorbance in the presence of the test sample and B represents the absorbance of the control reaction [26].

## 3 Results

### 3.1 LC-MS/MS Chromatography and Metabolite Identification

**Fig. 1:**
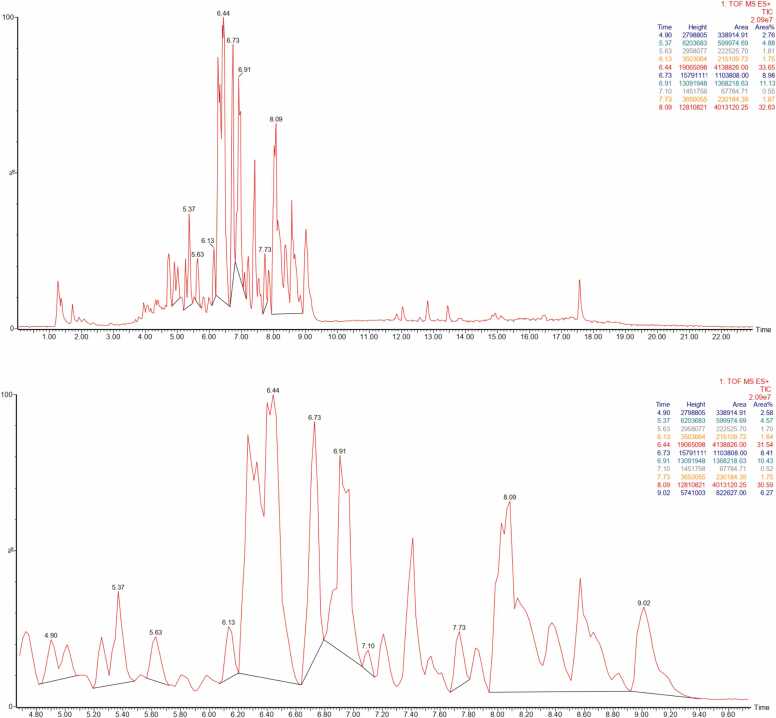
Total Ion Chromatogram (TIC) LC–MS/MS of *Mitragyna speciosa* Extract (ESI Positive Mode).

The LC–MS/MS analysis of the *Mitragyna speciosa* extract revealed a complex chemical profile dominated by alkaloid-type metabolites, as illustrated by the Total Ion Chromatogram (TIC) in Fig. 1. The TIC displayed numerous intense and well-resolved peaks concentrated mainly between retention times of approximately 5.0 and 9.5 min. This elution region is typical for semi-polar indole and oxindole alkaloids known to occur abundantly in *Mitragyna speciosa*. High signal intensity in this region indicates efficient ionization under positive electrospray ionization conditions. Chromatographic regions before 4 min and after 10 min exhibited relatively low peak intensities, suggesting a limited presence of highly polar or strongly nonpolar compounds. The clustering of peaks within a narrow retention window reflects structural similarities among the detected metabolites. Such similarities are consistent with shared indole-based core structures commonly reported for alkaloids of this plant. Sharp and symmetrical peak shapes indicate good chromatographic resolution. Adequate resolution is essential to reduce co-elution and improve compound annotation accuracy. The presence of both dominant and minor peaks highlights the chemical richness of the extract. Compounds present at lower abundance may still contribute to biological effects through additive interactions. The TIC profile indicates that the extraction method successfully enriched alkaloid constituents. Ethanolic solvents are known to effectively extract semi-polar alkaloids from plant matrices. The chromatographic distribution observed aligns with reported phytochemical characteristics of *Mitragyna speciosa*. This profile supports further interpretation of mass spectral data for metabolite identification.

Putative identification of compounds was performed using measured *m/z* values, predicted molecular formulas, and molecular weight matching, as presented in Table 1. Eleven compounds were tentatively identified in the *Mitragyna speciosa* extract. The detected metabolites were predominantly indole and oxindole alkaloids, which serve as chemotaxonomic markers of the genus. Mitrafoline was identified as the most abundant compound, contributing 31.54% of the relative composition. Mitragynine followed closely with a relative abundance of 30.59%. The prominence of mitragynine is consistent with previous reports identifying it as the major alkaloid in *Mitragyna speciosa*. Mitragynine has been extensively associated with analgesic and neuroactive properties. The high proportion of mitrafoline further emphasizes the alkaloid-dominant nature of the extract. Several compounds were detected at moderate levels, including corynoxine, methyl indoloquinolizine derivatives, and isopaynantheine. These alkaloids have been reported to exhibit diverse pharmacological activities. Minor constituents such as mitraphylline, corynantheidine, and 7-hydroxymitragynine were also detected. Although present at lower levels, these compounds remain biologically relevant. Notably, 7-hydroxymitragynine is reported to possess higher potency than mitragynine. The detection of multiple structurally related alkaloids illustrates the metabolic diversity of the plant.

**Table 1:** Putative Identification of Compounds in *Mitragyna speciosa* Extract Based on LC–MS/MS Analysis.

**No**	**Retention Time (min)**	***m/z* Measured**	**Molecular Weight (MW)**	**Molecular Formula**	**%Composition**	**Compound Name**
1	4.9	369.18	368.43	C_21_H_24_N_2_O_4_	2.58	Mitraphylline
2	5.37	371.20	370.44	C_20_H_26_N_4_O_3_	4.57	6-(9′-Purine-6′,8′-diyl)-2-beta-suberosanone
3	5.63	371.20	370.44	C_21_H_26_N_2_O_4_	1.70	Methyl (1′S,3R,4′R,4′aR,5′aS,10′aS)-2-hydroxy-1′-methyl-1′,3′,4′,4′a,5′,5′a,7′,8′,10′,10′a-decahydrospiro[indole-3,6′-pyrano[3,4-f]indolizine]-4′-carboxylate
4	6.13	399.19	398.45	C_22_H_26_N_2_O_5_	1.64	Methyl (1′S,3S,4′aS,5′aS,10′aR)-2-hydroxy-4-methoxy-1′-methyl-1′,4′a,5′,5′a,7′,8′,10′,10′a-octahydrospiro[indole-3,6′-pyrano[3,4-f]indolizine]-4′-carboxylate
5	6.44	401.21	400.47	C_22_H_28_N_2_O_5_	31.54	Mitrafoline
6	6.73	381.17	380.44	C_22_H_24_N_2_O_4_	8.41	Methyl 2-[(2E,3S)-3-ethyl-8-methoxy-3H,4H,6H,7H,12H-indolo[2,3-a]quinolizin-2-ylidene]-3-methoxyacrylate
7	6.91	385.21	384.47	C_22_H_28_N_2_O_4_	10.43	Corynoxine
8	7.10	415.22	414.50	C_23_H_30_N_2_O_5_	0.52	7-hydroxymitragynine
9	7.73	369.22	368.47	C_22_H_28_N_2_O_3_	1.75	Corynantheidine
10	8.09	399.23	398.5	C_23_H_30_N_2_O_4_	30.59	Mitragynine
11	9.02	397.21	396.4	C_23_H_28_N_2_O_4_	6.27	Isopaynantheine

Compound identification in this study is considered tentative because it relies on accurate mass measurements and database matching without confirmation using authentic reference standards. Despite this limitation, the identified compounds closely match phytochemical profiles previously reported for *Mitragyna speciosa*. Agreement between molecular weights, retention behavior, and known alkaloid structures supports the reliability of the LC–MS/MS analysis. Positive electrospray ionization proved effective for detecting nitrogen-containing alkaloids. This ionization mode facilitates protonation of indole alkaloids, resulting in strong and stable signals. The dominance of mitragynine and mitrafoline suggests their suitability as chemical markers for extract characterization. Marker compounds are essential for ensuring consistency in extract quality. Relative abundance data also indicate which constituents are most likely to contribute to biological activity. Highly abundant alkaloids are expected to play a primary role, while minor compounds may modulate these effects. The LC–MS/MS profiling establishes a chemical baseline for the extract. This baseline enables correlation between chemical composition and pharmacological activity. It also facilitates comparison with extracts obtained using different extraction methods. The generated data may support future quality control strategies. From a pharmacological perspective, the alkaloid-rich composition underscores the medicinal relevance of the plant. The presence of multiple bioactive alkaloids suggests a multitarget mode of action.

### 3.2 Physicochemical Properties and Predicted ADME Profiles

The selected compounds identified in *Mitragyna speciosa* extract (Table 2) represent a chemically diverse set of alkaloid-like scaffolds with structural features that are consistent with reported kratom metabolites. The dataset includes mitraphylline (MS1), mitrafoline (MS5), mitragynine (MS10), and 7-hydroxymitragynine (MS8), which are recognized as key constituents associated with the plant’s bioactivity. Several compounds share an indole-derived framework with fused ring systems, indicating high structural rigidity and a predominance of cyclic moieties. The SMILES representations confirm the presence of multiple heteroatoms, including nitrogen and oxygen, which are relevant for hydrogen bonding and target binding interactions. Ester functionalities are observed in several compounds, suggesting potential susceptibility to hydrolysis depending on biological conditions. Methoxy substituents are also present in multiple structures, which can influence lipophilicity and electronic distribution within aromatic systems. The 2D orientations illustrate stereochemical complexity, including multiple chiral centers that may affect binding selectivity and pharmacological profiles. Structural differences between mitragynine (MS10) and 7-hydroxymitragynine (MS8) are notable due to additional hydroxylation in MS8, which may alter polarity and receptor affinity. Corynoxine (MS7) and corynantheidine (MS9) exhibit closely related cores, supporting the presence of multiple analogs within the extract. The inclusion of purine-containing MS2 indicates that the extract may contain less typical nitrogenous metabolites or database-matched candidates with distinct heterocyclic motifs. The presence of spiro-type structures in MS3 and MS4 suggests additional complexity and potential variation in conformational behavior. Such scaffold diversity supports the possibility of multi-target biological activity. The structural dataset provides a rational basis for selecting compounds for docking, ADMET screening, or mechanistic evaluation. These structural features collectively indicate that the extract contains drug-like alkaloids with multiple interaction-capable functional groups.

**Table 2:** Chemical Structures and SMILES of Selected Compounds Identified in *Mitragyna speciosa* Extract.

**No**	**Compound Code**	**Compound Name**	**SMILES**	**Compound ID**	**2D Orientation**
1	MS1	Mitraphylline	C[C@H]1[C@H]2CN3CC[C@]4([C@@H]3C[C@@H]2 C(=CO1)C(=O)OC)C5 =CC=CC=C5NC4 =O	94160	
2	MS2	6-(9′-Purine-6′,8′-diyl)-2-beta-suberosanone	C[C@H]1CC[C@H]2[C@@H]3[C@@]1(CC2(C)C)[C@H](C(=O)C3)CN4C5 =C(C(=O)NC=N5)NC4 =O	135438508	
3	MS3	Methyl (1′S,3R,4′R,4′aR,5′aS,10′aS)-2-hydroxy-1′-methyl-1′,3′,4′,4′a,5′,5′a,7′,8′,10′,10′a-decahydrospiro[indole-3,6′-pyrano[3,4-f]indolizine]-4′-carboxylate	O=C([C@H]1CO[C@@H](C)[C@]([C@@]1([H])C[C@]23[H])([H])CN2CC[C@@]43 C(O)=NC5 =C4C=CC=C5)OC	(-)	
4	MS4	Methyl (1′S,3S,4′aS,5′aS,10′aR)-2-hydroxy-4-methoxy-1′-methyl-1′,4′a,5′,5′a,7′,8′,10′,10′a-octahydrospiro[indole-3,6′-pyrano[3,4-f]indolizine]-4′-carboxylate	O=C(C1 =CO[C@@H](C)[C@@]([C@]1([H])C[C@]23[H])([H])CN2CC[C@]43 C(O)=NC5 =C4C(OC)=CC=C5)OC	(-)	
5	MS5	Mitrafoline	CCC1CN2CCC3(C2CC1/C(=C/OC)/C(=O)OC)C4 =C(C=CC=C4O)NC3 =O	5379743	
6	MS6	Methyl 2-[(2E,3S)-3-ethyl-8-methoxy-3H,4H,6H,7H,12H-indolo[2,3-a]quinolizin-2-ylidene]-3-methoxyacrylate	CCC1CN2CCC3 =C(C2CC1C(=COC)C(=O)OC)NC4 =C3C(=CC=C4)OC	611919	
7	MS7	Corynoxine	CC[C@@H]1CN2CC[C@@]3([C@@H]2 C[C@@H]1/C(=C\OC)/C(=O)OC)C4 =CC=CC=C4NC3 =O	10475115	
8	MS8	7-hydroxymitragynine	CC[C@@H]1 CN2CC[C@]3(C(=NC4 =C3C(=CC=C4)OC)[C@@H]2C[C@@H]1/C(=C\OC)/C(=O)OC)O	44301524	
9	MS9	Corynantheidine	CC[C@@H]1 CN2CCC3 =C([C@@H]2C[C@@H]1/C(=C/OC)/C(=O)OC)NC4 =CC=CC=C34	6540753	
10	MS10	Mitragynine	CC[C@@H]1CN2CCC3 =C([C@@H]2C[C@@H]1/C(=C\OC)/C(=O)OC)NC4 =C3C(=CC=C4)OC	3034396	
11	MS11	Isopaynantheine	CO/C=C(\[C@H]1C[C@@H]2C3 =C(CCN2C[C@@H]1C=C)C4 =C(N3)C=CC=C4OC)/C(=O)OC	101804033	

The physicochemical descriptors in Table 3 indicate that the analyzed compounds fall within a relatively narrow molecular weight range, approximately 368–414 g/mol, which is generally compatible with oral drug-like space. All compounds satisfy Lipinski criteria with zero violations, supporting favorable drug-likeness characteristics for early-stage screening. The fraction of sp3 carbons ranges from 0.43 to 0.70, indicating a balance between aromaticity and three-dimensional saturation that may support binding specificity. The number of rotatable bonds varies from 2 to 6, suggesting that most compounds possess moderate flexibility while maintaining rigid polycyclic cores. Hydrogen bond acceptors range from 4 to 7, while donors range from 1 to 2, supporting the potential to form stabilizing polar interactions with protein targets. TPSA values span from 54.56 to 100.61 Å², indicating variability in polar surface exposure that can influence membrane permeability. MS2 shows the highest TPSA (100.61 Å²), which may reduce passive diffusion relative to lower-TPSA analogs. Consensus LogP values range from 1.82 to 3.21, reflecting moderate lipophilicity that can support absorption while still allowing aqueous handling. MS6 and MS10 exhibit higher lipophilicity, which is consistent with their lower predicted solubility values. Solubility classification based on ESOL and Ali models indicates that most compounds are predicted as soluble, while several are moderately soluble depending on the applied model. MS6 is consistently predicted to have the lowest solubility, aligning with its higher LogP and low ESOL solubility estimate. Differences among solubility predictors highlight the uncertainty of in silico solubility estimation and the need for experimental verification. Molar refractivity values are relatively high across compounds, indicating substantial molecular volume and polarizability. These physicochemical trends suggest that the compounds may exhibit good permeability but could face solubility limitations for the more lipophilic members. The results support prioritization of compounds with balanced LogP and TPSA for subsequent biological assays.

**Table 3:** Physicochemical Properties and Predicted Solubility of Selected *Mitragyna speciosa* Compounds.

**No**	**Property**	**MS1**	**MS2**	**MS3**	**MS4**	**MS5**	**MS6**
1	Formula	C_21_H_24_N_2_O_4_	C_20_H_26_N_4_O_3_	C_21_H_26_N_2_O_4_	C_22_H_26_N_2_O_5_	C_22_H_28_N_2_O_5_	C_23_H_30_N_2_O_4_
2	Molecular weight (g/mol)	368.43	370.45	370.44	398.45	400.47	398.50
3	Num. heavy atoms	27	27	27	29	29	29
4	Num. arom. heavy atoms	6	9	6	6	6	9
5	Fraction Csp3	0.52	0.7	0.62	0.55	0.55	0.52
6	Num. rotatable bonds	2	2	2	3	5	6
7	Num. H-bond acceptors	5	4	6	7	6	5
8	Num. H-bond donors	1	2	1	1	2	1
9	Molar Refractivity	106.47	102.87	108.61	114.63	115.42	116.89
10	TPSA (Å²)	67.87	100.61	71.36	80.59	88.10	63.79
11	Log *P*_o/w_ (iLOGP)	2.91	2.12	2.95	3.16	3.02	3.78
12	Log *P*_o/w_ (XLOGP3)	1.62	1.8	1.75	1.92	1.95	3.41
13	Log *P*_o/w_ (WLOGP)	1.11	2.08	1.68	1.96	1.45	3.12
14	Log *P*_o/w_ (MLOGP)	1.80	2.11	2.29	1.9	1.49	2.02
15	Log *P*_o/w_ (SILICOS-IT)	1.67	3.19	2.16	2.21	2.07	3.72
16	Consensus Log *P*_o/w_	1.82	2.26	2.17	2.23	2	3.21
17	Log *S* (ESOL)	-3.18	-3.39	-3.27	-3.48	-3.37	-4.29
18	Solubility	2.45e-01 mg/ml; 6.65e-04 mol/l	1.53e-01 mg/ml; 4.12e-04 mol/l	1.98e-01 mg/ml; 5.35e-04 mol/l	1.33e-01 mg/ml; 3.35e-04 mol/l	1.69e-01 mg/ml; 4.22e-04 mol/l	2.03e-02 mg/ml; 5.10e-05 mol/l
19	Class	Soluble	Soluble	Soluble	Soluble	Soluble	Moderately soluble
20	Log *S* (Ali)	-2.66	-3.53	-2.87	-3.24	-3.42	-4.43
21	Solubility	8.11e-01 mg/ml; 2.20e-03 mol/l	1.09e-01 mg/ml; 2.94e-04 mol/l	5.05e-01 mg/ml; 1.36e-03 mol/l	2.31e-01 mg/ml; 5.81e-04 mol/l	1.51e-01 mg/ml; 3.76e-04 mol/l	1.48e-02 mg/ml; 3.72e-05 mol/l
22	Class	Soluble	Soluble	Soluble	Soluble	Soluble	Moderately soluble
23	Log *S* (SILICOS-IT)	-3.96	-4.66	-3.5	-3.58	-3.89	-5
24	Solubility	4.01e-02 mg/ml; 1.09e-04 mol/l	8.10e-03 mg/ml; 2.19e-05 mol/l	1.18e-01 mg/ml; 3.18e-04 mol/l	1.04e-01 mg/ml; 2.60e-04 mol/l	5.11e-02 mg/ml; 1.28e-04 mol/l	3.95e-03 mg/ml; 9.91e-06 mol/l
25	Class	Soluble	Moderately soluble	Soluble	Soluble	Soluble	Moderately soluble
26	GI absorption	High	High	High	High	High	High
27	BBB permeant	No	No	Yes	No	No	Yes
28	P-gp substrate	Yes	Yes	No	No	Yes	No
29	CYP1A2 inhibitor	No	No	No	No	No	No
30	CYP2C19 inhibitor	No	No	No	No	No	No
31	CYP2C9 inhibitor	No	No	No	No	No	No
32	CYP2D6 inhibitor	Yes	Yes	Yes	Yes	Yes	Yes
33	CYP3A4 inhibitor	No	Yes	No	Yes	No	Yes
34	Log *K*_p_ (skin permeation) (cm/s)	-7.40	-7.28	-7.32	-7.37	-7.36	-6.31
35	Lipinski	Yes; 0 violation	Yes; 0 violation	Yes; 0 violation	Yes; 0 violation	Yes; 0 violation	Yes; 0 violation
36	Ghose	Yes	Yes	Yes	Yes	Yes	Yes
37	Veber	Yes	Yes	Yes	Yes	Yes	Yes
38	Egan	Yes	Yes	Yes	Yes	Yes	Yes
39	Muegge	Yes	Yes	Yes	Yes	Yes	Yes
40	Bioavailability Score	0.55	0.55	0.55	0.55	0.55	0.55
41	PAINS	0 alert	0 alert	0 alert	0 alert	0 alert	0 alert
42	Brenk	0 alert	0 alert	0 alert	0 alert	2 alerts: acyclic_C=C-O, michael_acceptor_1	1 alerts: acyclic_C=C-O, michael_acceptor_1
43	Leadlikeness	No; 1 violation: MW> 350	No; 1 violation: MW> 350	No; 1 violation: MW> 350	No; 1 violation: MW> 350	No; 1 violation: MW> 350	No; 1 violation: MW> 350
44	Synthetic accessibility	5.12	4.67	5.59	5.82	4.95	4.49

**No**	**Property**	**MS7**	**MS8**	**MS9**	**MS10**	**MS11**
1	Formula	C_22_H_28_N_2_O_4_	C_23_H_30_N_2_O_5_	C_22_H_28_N_2_O_3_	C_23_H_30_N_2_O_4_	C_23_H_28_N_2_O_4_
2	Molecular weight (g/mol)	384.47	414.49	368.47	398.50	396.48
3	Num. heavy atoms	28	30	27	29	29
4	Num. arom. heavy atoms	6	6	9	9	9
5	Fraction Csp3	0.55	0.57	0.5	0.52	0.43
6	Num. rotatable bonds	5	6	5	6	6
7	Num. H-bond acceptors	5	7	4	5	5
8	Num. H-bond donors	1	1	1	1	1
9	Molar Refractivity	113.39	121.14	110.39	116.89	116.41
10	TPSA (Å²)	67.87	80.59	54.56	63.79	63.79
11	Log *P*_o/w_ (iLOGP)	3.29	3.87	3.4	3.77	3.66
12	Log *P*_o/w_ (XLOGP3)	2.31	2.27	3.44	3.41	3.15
13	Log *P*_o/w_ (WLOGP)	1.75	1.92	3.11	3.12	2.9
14	Log *P*_o/w_ (MLOGP)	2.02	1.3	2.35	2.02	1.94
15	Log *P*_o/w_ (SILICOS-IT)	2.54	3.1	3.64	3.72	3.71
16	Consensus Log *P*_o/w_	2.38	2.49	3.19	3.21	3.07
17	Log *S* (ESOL)	-3.51	-3.59	-4.21	-4.29	-4.12
18	Solubility	1.19e-01 mg/ml; 3.11e-04 mol/l	1.06e-01 mg/ml; 2.56e-04 mol/l	2.28e-02 mg/ml; 6.19e-05 mol/l	2.03e-02 mg/ml; 5.10e-05 mol/l	3.03e-02 mg/ml; 7.65e-05 mol/l
19	Class	Soluble	Soluble	Moderately soluble	Moderately soluble	Moderately soluble
20	Log *S* (Ali)	-3.37	-3.6	-4.27	-4.43	-4.16
21	Solubility	1.63e-01 mg/ml; 4.23e-04 mol/l	1.04e-01 mg/ml; 2.52e-04 mol/l	1.99e-02 mg/ml; 5.41e-05 mol/l	1.48e-02 mg/ml; 3.72e-05 mol/l	2.75e-02 mg/ml; 6.93e-05 mol/l
22	Class	Soluble	Soluble	Moderately soluble	Moderately soluble	Moderately soluble
23	Log *S* (SILICOS-IT)	-4.48	-4.1	-4.9	-5	-4.66
24	Solubility	1.27e-02 mg/ml; 3.30e-05 mol/l	3.28e-02 mg/ml; 7.91e-05 mol/l	4.63e-03 mg/ml; 1.26e-05 mol/l	3.95e-03 mg/ml; 9.91e-06 mol/l	8.67e-03 mg/ml; 2.19e-05 mol/l
25	Class	Moderately soluble	Moderately soluble	Moderately soluble	Moderately soluble	Moderately soluble
26	GI absorption	High	High	High	High	High
27	BBB permeant	Yes	No	Yes	Yes	Yes
28	P-gp substrate	Yes	No	Yes	No	No
29	CYP1A2 inhibitor	No	No	No	No	No
30	CYP2C19 inhibitor	No	No	No	No	No
31	CYP2C9 inhibitor	No	No	No	No	Yes
32	CYP2D6 inhibitor	Yes	Yes	Yes	Yes	Yes
33	CYP3A4 inhibitor	Yes	Yes	Yes	Yes	Yes
34	Log *K*_p_ (skin permeation) (cm/s)	-7.01	-7.22	-6.11	-6.31	-6.48
35	Lipinski	Yes; 0 violation	Yes; 0 violation	Yes; 0 violation	Yes; 0 violation	Yes; 0 violation
36	Ghose	Yes	Yes	Yes	Yes	Yes
37	Veber	Yes	Yes	Yes	Yes	Yes
38	Egan	Yes	Yes	Yes	Yes	Yes
39	Muegge	Yes	Yes	Yes	Yes	Yes
40	Bioavailability Score	0.55	0.55	0.55	0.55	0.55
41	PAINS	0 alert	0 alert	0 alert: indol_3yl_alk	0 alert	0 alert
42	Brenk	2 alerts: acyclic_C=C-O, michael_acceptor_1	1 alerts: acyclic_C=C-O, michael_acceptor_1	1 alerts: acyclic_C=C-O, michael_acceptor_1	1 alerts: acyclic_C=C-O, michael_acceptor_1	2 alerts: acyclic_C=C-O, isolated_alkene, michael_acceptor_1
43	Leadlikeness	No; 1 violation: MW> 350	No; 1 violation: MW> 350	No; 1 violation: MW> 350	No; 1 violation: MW> 350	No; 1 violation: MW> 350
44	Synthetic accessibility	4.78	5.21	4.27	4.49	4.5

The predicted pharmacokinetic properties indicate high gastrointestinal absorption for all evaluated compounds, suggesting favorable potential for oral exposure. BBB permeability predictions vary across the dataset, with several compounds predicted as BBB permeant, including MS3, MS6, MS7, MS9, MS10, and MS11, which may relate to central nervous system activity. MS1, MS2, MS4, MS5, and MS8 are predicted as non-BBB permeant, indicating potential restriction to peripheral exposure. P-glycoprotein substrate predictions differ among compounds, which may influence efflux susceptibility and bioavailability. Several compounds are predicted as P-gp substrates, implying that transporter-mediated efflux could reduce intracellular accumulation in some tissues. CYP inhibition profiles indicate broad CYP2D6 inhibition across all compounds, which may represent a potential risk for drug–drug interactions. CYP3A4 inhibition is predicted for multiple compounds, including MS2, MS4, MS6, and the later set MS7–MS11, further emphasizing interaction potential. No inhibition is predicted for CYP1A2, CYP2C19, and CYP2C9 in most cases, although MS11 shows CYP2C9 inhibition. Bioavailability scores are uniformly reported as 0.55, suggesting moderate predicted oral bioavailability across the series. PAINS alerts are absent across compounds, supporting low risk of promiscuous assay interference based on this filter. Brenk alerts appear in several compounds due to Michael acceptor-related motifs, which may indicate reactive substructures requiring careful interpretation in screening assays. Lead-likeness evaluation indicates one violation for all compounds due to molecular weight exceeding 350 g/mol, which may affect optimization considerations. Synthetic accessibility scores range from approximately 4.27–5.82, indicating moderate synthetic complexity consistent with polycyclic natural product scaffolds. Predicted skin permeation values (LogKp) suggest limited transdermal penetration for most compounds, with slightly higher permeability predicted for more lipophilic structures. These ADME patterns support the feasibility of oral exploration while highlighting metabolic and transporter-related considerations. The in silico profiling provides a useful framework for selecting candidates for docking and biological validation with attention to solubility and CYP interaction risks.

### 3.3 Target Prediction and Candidate Selection

The in silico target prediction results for selected compounds from *Mitragyna speciosa* provide an initial mechanistic overview of potential protein interactions that may contribute to the extract’s biological effects (Table 4). The predicted targets span diverse protein classes, including receptors, enzymes, kinases, transporters, and ion channels, indicating a broad pharmacological space. Mitraphylline (MS1) exhibits the widest target spectrum, suggesting a multi-target interaction profile that may support pleiotropic activity. Nicotinic acetylcholine receptor subunits CHRNA4 and CHRNB2 are predicted targets of MS1, supporting possible modulation of neuronal signaling pathways. Opioid-related targets such as OPRM1, OPRD1, and OPRK1 are also listed for MS1, which may be relevant to neuromodulatory and analgesic mechanisms. Ion channel-associated proteins including HCN1, HCN4, and SCN5A appear among the predicted targets, indicating potential effects on membrane excitability and electrical conductance. Several key signaling kinases, including JAK1, JAK2, JAK3, AKT1, and PIK3CA, are predicted for MS1, which may relate to immune signaling and cell survival regulation. Growth factor receptors such as EGFR, MET, ERBB2, and FGFR1 are included, suggesting potential links to proliferation-related pathways. Cell-cycle regulators including CDK1, CDK2, CDK5, CDK9, CCNA1, CCNA2, and CHEK1 are also predicted, implying possible modulation of cell-cycle control. DNA repair-associated proteins PARP1 and PARP2 further indicate potential involvement in stress response signaling. ABCB1 appears as a predicted transporter target, which may influence intracellular exposure and distribution. Protease-related targets such as CTSK and PRSS1 suggest potential relevance to proteolytic activity and inflammatory processes. This wide target diversity supports the selection of MS1 for deeper validation using molecular docking and functional assays. These findings support the possibility that multiple pathways contribute to the biological activity of the extract.

**Table 4:** Predicted Molecular Targets of Selected *Mitragyna speciosa* Compounds.

**No**	**Compound Code**	**Compound Name**	**Target Prediction**
1	MS1	Mitraphylline	CHRNA4, CHRNB2, ADRA1B, DPP4, TERT, HCN4, HCN1, HTR1F, HTR1D, JAK3, HRH3, PDE10A, EGFR, PLD1, PLD2, DRD5, ADRB2, HTR5A, DPP9, GPR142, MPO, PRKCQ, CDC25C, PDE9A, DPP7, PDE1C, DPP8, SCN5A, ABCB1, PDE2A, LCK, PARP1, BDKRB2, CCNE1, CDK2, CDK9, CCNT1, ADRA1D, CCR2, MAPK8, MAPK10, PDE8B, SSTR5, ADORA1, FAP, CTSK, MTOR, PIK3CA, JAK2, TGFBR1, SLC6A9, JAK1, AKT1, PREP, MET, OPRM1, OPRD1, CHRNA4, ACKR3, F2, PIM1, PIM2, PARP2, OPRK1, CFD, BRPF1, KCNJ1, MTNR1B, HRH2, LTA4H, CCNA1, CCNA2, ROCK2, ROCK1, HRH4, HTR4, HTR1E, PRKCD, ALK, KCNA5, NPY5R, F10, IRAK4, HIF1A, FLT1, CDK5R1, CDK5, RET, PRSS1, FGFR1, CHEK1, WDR5, IKBKB, SRC, MALT1, TYRO3, SLC18A2, SLC6A4, ERBB2, MAPKAPK2, dan CDK1.
2	MS2	6-(9′-Purine-6′,8′-diyl)-2-beta-suberosanone	ACHE, BRD3, ADORA1, ADORA2B, ADORA3, BRD4, BRDT, ADORA2A, PDE4A, MTNR1A, MTNR1B, BRD2, HTR2C, FKBP5, HSP90AA1, CTSK, IGF1R, ITK, PPARG, NQO2, NAAA, P2RX7, NR3C1, ROCK2, ROCK1, CYP19A1, NTRK1, S1PR1, CCR1, CNR2, GAA, HSD11B1, HSP90AB1, PDE5A, EDNRA, PDE4B, PDE4D, PDE4C, SLC6A2, MMP9, MMP2, CYP1A2, ALDH2, ESR1, DBF4, CDC7, ESR2, RPS6KB1, AR, GSK3B, PKN2, CTSL, AURKA, KCNH2, MMP3, MMP1, CYP2C19, CACNA1B, PRMT3, CHIA, EPHX2, TTR, PARP2, PSEN2, PSENEN, NCSTN, APH1A, PSEN1, APH1B, CA7, CA12, CA9, KCNA5, MAPKAPK2, RPS6KA3, HDAC8, NR3C2, CCNC, CDK8, ABCB1, ABCG2, FKBP1A, PDE3A, PDE3B, NPY5R, CTSS, CTSV, PRKCQ, CTSB, TRPV1, CHRM4, EPHX1, CFD, C5AR1, ADAM17, AURKB, CA6, CA14, CA13, CA5B, FFAR1, CA5A, HPGD, CCR5, dan GPR139.
3	MS3	Methyl (1′S,3R,4′R,4′aR,5′aS,10′aS)-2-hydroxy-1′-methyl-1′,3′,4′,4′a,5′,5′a,7′,8′,10′,10′a-decahydrospiro[indole-3,6′-pyrano[3,4-f]indolizine]-4′-carboxylate	(-)
4	MS4	Methyl (1′S,3S,4′aS,5′aS,10′aR)-2-hydroxy-4-methoxy-1′-methyl-1′,4′a,5′,5′a,7′,8′,10′,10′a-octahydrospiro[indole-3,6′-pyrano[3,4-f]indolizine]-4′-carboxylate	(-)
5	MS5	Mitrafoline	(-)
6	MS6	Methyl 2-[(2E,3S)-3-ethyl-8-methoxy-3H,4H,6H,7H,12H-indolo[2,3-a]quinolizin-2-ylidene]-3-methoxyacrylate	(-)
7	MS7	Corynoxine	CHRNA4, CHRNB2, PDE10A, HCN4, HCN1, OPRD1, TERT, HRH3, SCN5A, DPP9, OPRK1, OPRM1, DPP8, BRPF1, PARP1, PDE2A, PARP2, DPP7, SLC6A4, ADORA1, CDK5R1, CDK5, MET, TRPA1, PDE9A, PDE1C, HTR1F, EGFR, JAK3, JAK2, TGFBR1, KDM1A, GPR142, PLD1, PLD2, SSTR5, HRH2, HTR5A, KCNJ1, CDK1, ALK, AURKA, OPRL1, ITK, PRKCQ, SLC22A2, SLC47A1, CCND1, CDK4, PRSS1, HTR3B, HTR3A, POLR1A, PDE8B, HTR4, CFD, HTR1E, UTS2R, ATM, CDC25C, MTOR, BDKRB2, PRKCB, IKBKB, SLC6A2, DRD5, MAPK8, MAPK10, PIK3CA, CHEK1, HTR7, MAOA, ADAM17, ERG, CCNE1, CDK2, CCNE2, CCNT1, CDK9, ELANE, LIMK1, LIMK2, CCNA2, CCNA1, PREP, FAP, ATAD2, PRCP, PIM1, GSK3B, JAK1, PIM2, CASP7, CACNA1G, RET, SYK, PRKCZ, MALT1, NQO1, HRH4, BIRC3, dan ADRB1.
8	MS8	7-hydroxymitragynine	(-)
9	MS9	Corynantheidine	BCHE, OPRD1, HTR1A, ADRA2A, ADRA2C, HTR2A, ADRA2B, DRD2, HTR6, DRD3, ADRA1A, ADRA1B, ADORA3, HTR2B, ADRA1D, CYP2D6, OPRM1, KIF11, PDE3A, PDE7A, CMA1, PREP, CHEK1, SLC6A3, DRD1, SLC6A4, OPRL1, MAPK14, HCRTR2, HCRTR1, CACNA1H, TACR3, LCK, HTR5A, CDK2, CCNA1, CCNA2, HTR7, CXCR2, MAPK11, SMO, LRRK2, PIK3CA, CAPN1, CAPNS1, SLC6A9, SYK, TNKS2, TNKS, WNT3A, SLC27A1, FAAH, ELOVL6, ABCB1, SLC5A2, KCNA3, SLC5A1, RHOA, MDM4, MAPK8, FASN, LIMK2, ACHE, NOX4, TRPV1, BDKRB1, RIPK1, BRD2, CRHR1, F2, PLK1, BRPF1, PTK2B, HPGDS, RBP4, IRAK4, AVPR2, NR1H4, PDE2A, CPT2, BRD4, ITK, ABL1, CDK5R1, CDK5, PDGFRA, PDGFRB, NAMPT, SSTR3, ERBB2, CYP11B1, CAPN2, JAK3, JAK1, JAK2, WNT3, PGGT1B, FNTA, AURKB, SOAT1, CETP, GCK, ABCG2, NR3C2, dan NTRK1.
10	MS10	Mitragynine	OPRD1, BCHE, ADRA2A, ADRA2C, DRD2, ADRA2B, HTR1A, dan HTR2A.
11	MS11	Isopaynantheine	OPRD1, BCHE, ADRA2A, ADRA2C, dan ADRA2B.

MS2, identified as 6-(9′-Purine-6′,8′-diyl)-2-beta-suberosanone, also demonstrates an extensive predicted target profile involving neuroactive receptors, signaling enzymes, and regulatory proteins. Neurotransmission-associated targets such as ACHE, adenosine receptors (ADORA1, ADORA2A, ADORA2B, ADORA3), and serotonin receptor HTR2C indicate potential neuromodulatory relevance. The predicted involvement of ESR1, ESR2, and AR suggests that MS2 may interact with hormone-related signaling pathways that influence metabolic and cellular regulation. Multiple phosphodiesterases including PDE4A, PDE4B, PDE4C, PDE4D, PDE5A, PDE3A, and PDE3B are predicted, indicating potential modulation of cyclic nucleotide signaling. Matrix metalloproteinases such as MMP1, MMP2, MMP3, and MMP9 are included, supporting possible associations with inflammation and extracellular matrix remodeling. Kinase-related targets including IGF1R, GSK3B, RPS6KB1, and MAPKAPK2 suggest potential regulation of growth and survival pathways. Epigenetic regulators BRD2, BRD3, BRD4, BRDT, and HDAC8 are predicted, implying that MS2 may influence transcriptional regulation. Transporters ABCB1 and ABCG2 are also present, suggesting that efflux mechanisms may affect intracellular accumulation. Drug metabolism-associated enzymes such as CYP1A2 and CYP2C19 highlight potential metabolic interaction considerations. Corynoxine (MS7) shows an extensive target spectrum overlapping with MS1, including opioid receptors and multiple kinase targets. Corynantheidine (MS9), mitragynine (MS10), and isopaynantheine (MS11) share predicted interactions with OPRD1 and adrenergic receptors, supporting receptor-level mechanisms related to neuroactivity. Several compounds, including MS3, MS4, MS5, MS6, and MS8, show no predicted targets, which may reflect database limitations or insufficient matching during the prediction step. The repeated presence of opioid, adrenergic, and neurotransmission-related targets suggests that central signaling pathways may represent a major mechanistic direction for the extract. Experimental validation remains necessary to confirm binding and functional effects at the predicted targets.

### 3.4 Common Target Identification and PPI Network Analysis

**Fig. 2:**
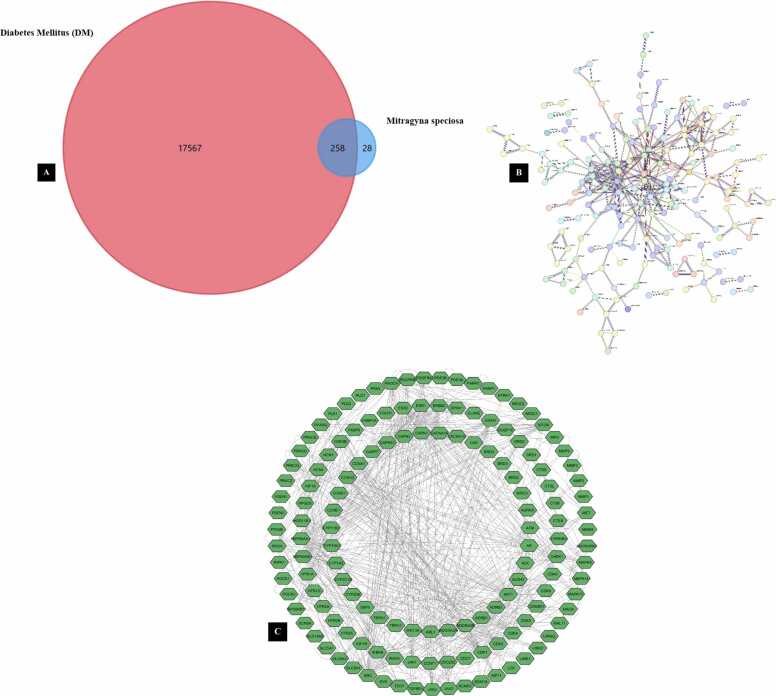
Network Pharmacology Analysis of *Mitragyna Speciosa* against Diabetes Mellitus: (A) Venn Diagram of Shared Targets, (B) PPI Network of Common Targets, And (C) Interaction Network Visualization.

The target overlap analysis between *Mitragyna speciosa* and diabetes mellitus is visualized in Fig. 2**A**, revealing a subset of shared molecular targets that may explain the potential antidiabetic relevance of this plant. The Venn diagram indicates that diabetes mellitus is associated with a very large target pool, whereas *Mitragyna speciosa* is linked to a more limited number of predicted targets. Based on the visualization, diabetes mellitus involved 17,567 targets, *Mitragyna speciosa* included 258 targets, and 28 targets were identified as common targets between both datasets. This overlap suggests that the potential therapeutic effects of *Mitragyna speciosa* may be mediated through a focused group of proteins that are directly involved in diabetes pathogenesis. Although the number of shared targets is relatively small compared to the overall disease-related target list, these common targets are expected to be biologically meaningful. Such shared targets may represent proteins regulating glucose metabolism, insulin signaling, oxidative stress responses, inflammatory cascades, and vascular dysfunction, which are key features of diabetes mellitus. The identification of 28 common targets provides a rational basis for subsequent network construction and hub gene prioritization. This approach supports the concept that herbal medicines exert therapeutic actions through multitarget modulation rather than a single isolated mechanism. The overlap results also serve as a filtering step to reduce the complexity of disease targets into a manageable and disease-relevant subset for downstream analyses. The shared targets enable the establishment of a mechanistic bridge between the predicted compound targets and diabetes-associated proteins. This strategy improves the interpretability of network pharmacology outputs by focusing on the intersection of compound-related and disease-related biology. The overlap information further supports the selection of candidate proteins for molecular docking and pathway enrichment studies. In addition, the shared targets may represent convergent nodes where multiple signaling pathways intersect, increasing their relevance as potential therapeutic regulators. The overlap analysis therefore provides a foundational framework for exploring the systemic mechanisms of *Mitragyna speciosa* in diabetes mellitus. These findings support the continuation of network-based analyses to identify key regulatory proteins and pathways.

The shared targets were further analyzed through protein–protein interaction mapping, as shown in Fig. 2**B**, which demonstrates a complex interaction landscape among diabetes-relevant proteins influenced by *Mitragyna speciosa*. The PPI visualization shows a dense and highly interconnected network, suggesting extensive molecular communication among the common targets. The presence of a tightly connected central region implies that several proteins may act as hubs with high connectivity and regulatory importance. Hub proteins in such networks often coordinate signaling events across inflammation, metabolism, and cellular stress responses, which are core processes involved in diabetes progression. The interaction density supports the hypothesis that modulation of one target may influence other targets indirectly through network propagation effects. This property is particularly relevant in diabetes mellitus, which is a multifactorial disease characterized by simultaneous dysregulation of metabolic and inflammatory pathways. A complementary interaction network visualization is provided in Fig. 2**C**, presenting the shared targets in a circular layout with extensive cross-linking that highlights the systemic nature of the underlying biological processes. A highly connected network structure may also indicate that these targets participate in overlapping pathways such as PI3K–AKT signaling, MAPK cascades, cytokine-mediated responses, and receptor-regulated metabolic control. From a network pharmacology perspective, the observed connectivity supports the plausibility of multitarget actions for *Mitragyna speciosa* metabolites. The PPI network can be used to prioritize key proteins using topological parameters such as degree, betweenness centrality, and closeness centrality. Proteins with higher topological importance may represent strategic intervention points and suitable candidates for molecular docking validation. This network-driven prioritization reduces bias in target selection and enhances the mechanistic value of subsequent computational steps. The PPI results also strengthen the rationale for conducting enrichment analyses to identify dominant biological processes and pathways associated with the shared targets. Furthermore, the network architecture suggests that *Mitragyna speciosa* may exert broad therapeutic potential by influencing interconnected proteins rather than acting on a single enzyme or receptor. The network analysis provides a mechanistic scaffold for integrating compound–target information with disease biology. These findings support further investigation of hub targets and pathway enrichment to clarify the principal antidiabetic mechanisms of *Mitragyna speciosa*.

### 3.5 Hub Target Identification and MCC Ranking Analysis

**Fig. 3:**
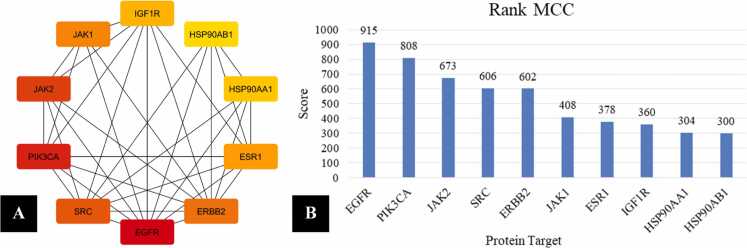
Hub Target Analysis of Common Targets Between *Mitragyna Speciosa* and Diabetes Mellitus: (A) Interaction Network of Top Hub Targets and (B) MCC Ranking Scores of the Selected Hub Proteins.

Hub target identification using the MCC algorithm revealed a set of highly connected proteins that may act as key regulatory nodes linking *Mitragyna speciosa* to diabetes mellitus, and the interaction relationships among these hubs are visualized in Fig. 3**A**. The network shows strong interconnectivity among the selected targets, indicating that these proteins participate in coordinated signaling rather than functioning as isolated components. EGFR appears as the most dominant hub based on its highest MCC score (915), suggesting a central regulatory position within the shared target network. The prominence of EGFR may be relevant to diabetes-associated cellular stress responses, inflammatory signaling, and tissue remodeling processes that contribute to metabolic dysfunction. PIK3CA ranked second (808), highlighting the importance of PI3K-mediated signaling in glucose homeostasis, insulin sensitivity, and metabolic regulation. JAK2 (673) and SRC (606) were also identified as major hubs, supporting the involvement of cytokine-associated signaling and tyrosine kinase activity in diabetes-related molecular mechanisms. ERBB2 (602) emerged as another high-ranking hub, indicating that receptor tyrosine kinase crosstalk may contribute to downstream metabolic and inflammatory pathway modulation. The presence of multiple receptor-associated and intracellular kinases among the top-ranked proteins supports a signaling-centered mechanism of action for the shared targets. A tightly connected hub structure also implies that perturbation of one node may propagate functional changes across other proteins within the system. This property is consistent with the multifactorial nature of diabetes mellitus, which involves overlapping inflammatory and metabolic disturbances. The hub connectivity suggests that multitarget modulation by plant-derived metabolites may be more effective than single-target intervention. Such a pattern supports the network pharmacology concept that complex diseases can be influenced through coordinated regulation of multiple interacting nodes. The hub targets identified from this network represent strategic candidates for downstream validation using molecular docking and pathway enrichment analyses. These results provide a mechanistic basis for prioritizing proteins that may mediate the biological effects of *Mitragyna speciosa* in diabetes mellitus.

The MCC ranking distribution of the selected hub proteins is shown in Fig. 3**B**, providing quantitative prioritization of the most influential nodes within the shared target network. EGFR remained the top-ranked target with the highest MCC score (915), reinforcing its potential role as a primary regulatory hub. PIK3CA followed with an MCC score of 808, confirming the central relevance of PI3K signaling in metabolic control and insulin-related pathways. JAK2 (673) ranked higher than SRC (606) and ERBB2 (602), suggesting that cytokine-driven signaling may have a particularly strong influence within the shared diabetes-related network. JAK1 displayed a moderate MCC score (408), indicating that the JAK/STAT axis is represented by multiple nodes that may cooperate in inflammatory regulation. ESR1 ranked next (378), supporting the contribution of estrogen receptor signaling to metabolic regulation, insulin responsiveness, and lipid homeostasis. IGF1R also appeared as a hub target (360), which is consistent with its role in insulin-like growth factor signaling and glucose uptake regulation. Two molecular chaperones, HSP90AA1 (304) and HSP90AB1 (300), were included among the top-ranked targets, indicating potential involvement of proteostasis and stress-response mechanisms. The appearance of HSP90 family proteins may reflect adaptive cellular responses to oxidative stress and protein misfolding associated with chronic metabolic disorders. The combined presence of receptor tyrosine kinases, intracellular kinases, hormone receptors, and stress-response proteins suggests that multiple pathways converge within the hub target set. These pathways are closely linked to insulin signaling efficiency, inflammatory burden, and metabolic tissue function. The prioritization pattern provides a rational framework for selecting protein targets for docking analysis with *Mitragyna speciosa* metabolites. High-ranked hub proteins are expected to be more influential in network stability and may yield stronger mechanistic relevance when validated experimentally. The MCC-based hub analysis therefore strengthens the interpretation that *Mitragyna speciosa* may exert antidiabetic potential through integrated modulation of interconnected signaling networks.

### 3.6 GO and KEGG Enrichment Analysis

**Fig. 4:**
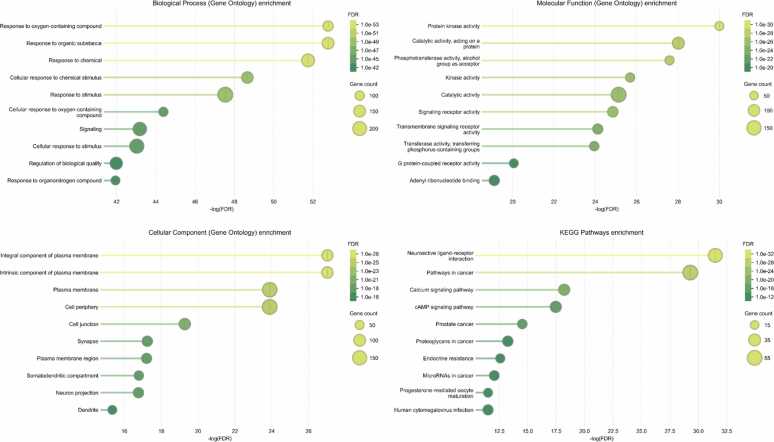
Functional Enrichment Analysis of Common Targets Between *Mitragyna Speciosa* and Diabetes Mellitus: GO Enrichment for Biological Process, Molecular Function, Cellular Component, and KEGG Pathway Enrichment.

Functional enrichment analysis provides a mechanistic interpretation of the shared targets between *Mitragyna speciosa* and diabetes mellitus (Fig. 4). In the GO Biological Process category, the most enriched terms were dominated by response-related processes, including response to oxygen-containing compounds, response to organic substances, and response to chemicals, indicating that the shared targets are strongly associated with cellular adaptation to metabolic and oxidative stress. The enrichment of cellular response to chemical stimulus and cellular response to stimulus further supports the involvement of stress-responsive signaling networks that are commonly activated during hyperglycemia and chronic inflammation. Signaling-related biological processes were also enriched, suggesting that the common targets participate in coordinated intracellular communication rather than isolated metabolic events. The presence of regulation of biological quality implies potential roles in maintaining cellular homeostasis, which is often disrupted in diabetes through oxidative damage and inflammatory burden. In the GO Molecular Function category, protein kinase activity and catalytic activity acting on a protein were among the top enriched terms, highlighting the importance of phosphorylation-driven signaling in the shared target network. The enrichment of kinase activity, phosphotransferase activity, and transferase activity indicates that enzymatic regulation through phosphorylation may represent a key mechanism connecting the plant targets to diabetes-associated pathways. Signaling receptor activity and transmembrane signaling receptor activity were also enriched, supporting the relevance of receptor-mediated pathway initiation and downstream cascade activation. The appearance of G protein-coupled receptor activity suggests potential involvement of neuroendocrine and metabolic receptor systems that can regulate glucose metabolism and insulin sensitivity. Adenyl ribonucleotide binding enrichment further reflects the participation of energy-related and nucleotide-dependent enzymatic functions in the shared target set. These GO results indicate that the shared targets are enriched in stress response, receptor signaling, and kinase-centered regulatory mechanisms that are central to diabetes pathophysiology.

The GO Cellular Component enrichment showed that the shared targets were mainly localized to membrane-associated regions, including the integral component of plasma membrane, intrinsic component of plasma membrane, and plasma membrane itself. This localization pattern supports the biological relevance of receptor and transporter functions, as many diabetes-associated signaling events begin at the cell surface through ligand–receptor interactions. Enrichment in cell periphery and plasma membrane region further indicates that the shared targets are positioned at key interfaces where extracellular signals are converted into intracellular responses. The enrichment of cell junction suggests possible involvement in intercellular communication and barrier regulation, which are important in vascular complications of diabetes. Synapse, neuron projection, dendrite, and somatodendritic compartment enrichment suggests that neuronal and neuroendocrine signaling components may be involved, aligning with the known relationship between metabolic regulation and neural control of appetite, insulin secretion, and peripheral glucose utilization. In the KEGG pathway enrichment results, neuroactive ligand–receptor interaction emerged as the top pathway, supporting the potential contribution of receptor-mediated neuroendocrine mechanisms in the shared target network. Pathways in cancer were also highly enriched, which may reflect the presence of central growth and survival signaling nodes such as EGFR, PI3K, and related kinases that are shared between diabetes-related dysregulation and proliferative signaling pathways. Calcium signaling pathway and cAMP signaling pathway enrichment further reinforces the importance of second messenger systems in insulin secretion, glucose transport, and metabolic homeostasis. Endocrine resistance and progesterone-mediated oocyte maturation enrichment may indicate broader hormonal signaling involvement, which can influence metabolic balance and insulin responsiveness. Proteoglycans in cancer and microRNAs in cancer pathways suggest that extracellular matrix regulation and post-transcriptional control mechanisms may also contribute to the shared biological landscape. Human cytomegalovirus infection enrichment is commonly observed in enrichment outputs due to shared immune and signaling proteins and may reflect inflammation-related components rather than direct viral involvement. The combined GO and KEGG enrichment patterns indicate that *Mitragyna speciosa* may influence diabetes mellitus through integrated modulation of membrane-associated receptors, kinase-driven signaling cascades, and second messenger pathways linked to metabolic and inflammatory regulation. These enrichment findings provide a strong rationale for prioritizing hub targets and pathways for molecular docking and experimental validation to clarify the principal antidiabetic mechanisms of *Mitragyna speciosa*.

### 3.7 In Silico Toxicity Prediction and Safety Assessment

The in silico toxicity prediction results provide an early safety screening profile for the selected *Mitragyna speciosa* compounds and highlight several endpoints that require careful interpretation before biological translation (Table 5). Hepatotoxicity was predicted as inactive for all compounds, suggesting a low likelihood of direct liver toxicity based on this computational model. Cardiotoxicity, mutagenicity, and cytotoxicity were also consistently predicted as inactive across the series, indicating no strong alerts for these major safety concerns. In contrast, respiratory toxicity was predicted as active for all compounds, which represents a uniform warning signal that should be considered during further preclinical planning. Clinical toxicity was also predicted as active across all compounds, indicating that adverse outcome potential may exist despite the absence of mutagenicity or cytotoxicity flags. Neurotoxicity predictions differed among compounds, where MS1, MS2, and MS3 were predicted as active, while MS4, MS5, and MS6 were inactive, showing that neurotoxicity risk may be compound-dependent. Nephrotoxicity was predicted as active for MS3, MS5, and MS6, indicating potential kidney-related concerns for specific members of the set. Carcinogenicity showed variable predictions, with MS1, MS4, and MS6 predicted as active, while MS2, MS3, and MS5 were inactive, suggesting that carcinogenicity risk is not uniform across the compounds. Immunotoxicity was predicted as active for MS3 and MS4, while remaining compounds were inactive, indicating that immune-related toxicity may be associated with particular structural features. BBB-barrier prediction was active for MS1, MS2, MS3, MS4, and MS6, whereas MS5 was inactive, implying that most compounds may cross the blood–brain barrier and potentially contribute to central nervous system exposure. Ecotoxicity was predicted as active for MS3, MS4, and MS6, indicating that environmental toxicity considerations may arise depending on compound selection. Nutritional toxicity was predicted as active for MS1, MS3, MS4, and MS5, while MS2 and MS6 were inactive, reflecting differences in predicted systemic disturbance profiles. Receptor-associated toxicity endpoints such as AhR, AR, AR-LBD, aromatase, ER, ER-LBD, and PPAR-gamma were consistently predicted as inactive, suggesting limited endocrine disruption potential based on these markers. Stress response and genomic stability indicators, including nrf2/ARE, HSE, MMP, p53, and ATAD5, were also inactive across the compounds, supporting the absence of strong predicted oxidative-stress or DNA-damage pathway activation. Thyroid-related targets (THRα, THRβ) and transthyretin (TTR) were inactive, indicating low predicted interference with thyroid hormone signaling and transport. These findings suggest that the compounds may exhibit acceptable profiles in several critical toxicity categories, while still presenting notable alerts for respiratory and clinical toxicity that must be validated experimentally.

**Table 5:** In Silico Toxicity Prediction Profiles of Selected *Mitragyna speciosa* Compounds.

**No**	**Target**	**MS1**	**MS2**	**MS3**	**MS4**	**MS5**	**MS6**
1	Hepatotoxicity	Inactive	Inactive	Inactive	Inactive	Inactive	Inactive
2	Neurotoxicity	Active	Active	Active	Inactive	Inactive	Inactive
3	Nephrotoxicity	Inactive	Inactive	Active	Inactive	Active	Active
4	Respiratory toxicity	Active	Active	Active	Active	Active	Active
5	Cardiotoxicity	Inactive	Inactive	Inactive	Inactive	Inactive	Inactive
6	Carcinogenicity	Active	Inactive	Inactive	Active	Inactive	Active
7	Immunotoxicity	Inactive	Inactive	Active	Active	Inactive	Inactive
8	Mutagenicity	Inactive	Inactive	Inactive	Inactive	Inactive	Inactive
9	Cytotoxicity	Inactive	Inactive	Inactive	Inactive	Inactive	Inactive
10	BBB-barrier	Active	Active	Active	Active	Inactive	Active
11	Ecotoxicity	Inactive	Inactive	Active	Active	Inactive	Active
12	Clinical toxicity	Active	Active	Active	Active	Active	Active
13	Nutritional toxicity	Active	Inactive	Active	Active	Active	Inactive
14	Aryl hydrocarbon Receptor (AhR)	Inactive	Inactive	Inactive	Inactive	Inactive	Inactive
15	Androgen Receptor (AR)	Inactive	Inactive	Inactive	Inactive	Inactive	Inactive
16	Androgen Receptor Ligand Binding Domain (AR-LBD)	Inactive	Inactive	Inactive	Inactive	Inactive	Inactive
17	Aromatase	Inactive	Inactive	Inactive	Inactive	Inactive	Inactive
18	Estrogen Receptor Alpha (ER)	Inactive	Inactive	Inactive	Inactive	Inactive	Inactive
19	Estrogen Receptor Ligand Binding Domain (ER-LBD)	Inactive	Inactive	Inactive	Inactive	Inactive	Inactive
20	Peroxisome Proliferator Activated Receptor Gamma (PPAR-Gamma)	Inactive	Inactive	Inactive	Inactive	Inactive	Inactive
21	Nuclear factor (erythroid-derived 2)-like 2/antioxidant responsive element (nrf2/ARE)	Inactive	Inactive	Inactive	Inactive	Inactive	Inactive
22	Heat shock factor response element (HSE)	Inactive	Inactive	Inactive	Inactive	Inactive	Inactive
23	Mitochondrial Membrane Potential (MMP)	Inactive	Inactive	Inactive	Inactive	Inactive	Inactive
24	Phosphoprotein (Tumor Supressor) p53	Inactive	Inactive	Inactive	Inactive	Inactive	Inactive
25	ATPase family AAA domain-containing protein 5 (ATAD5)	Inactive	Inactive	Inactive	Inactive	Inactive	Inactive
26	Thyroid hormone receptor alpha (THRα)	Inactive	Inactive	Inactive	Inactive	Inactive	Inactive
27	Thyroid hormone receptor beta (THRβ)	Inactive	Inactive	Inactive	Inactive	Inactive	Inactive
28	Transtyretrin (TTR)	Inactive	Inactive	Inactive	Inactive	Inactive	Inactive
29	Ryanodine receptor (RYR)	Inactive	Inactive	Inactive	Inactive	Inactive	Inactive
30	GABA receptor (GABAR)	Inactive	Active	Inactive	Inactive	Inactive	Inactive
31	Glutamate N-methyl-D-aspartate receptor (NMDAR)	Inactive	Inactive	Inactive	Inactive	Inactive	Inactive
32	alpha-amino-3-hydroxy-5-methyl-4-isoxazolepropionate receptor (AMPAR)	Inactive	Inactive	Inactive	Inactive	Inactive	Inactive
33	Kainate receptor (KAR)	Inactive	Inactive	Inactive	Inactive	Inactive	Inactive
34	Achetylcholinesterase (AChE)	Active	Inactive	Active	Active	Active	Inactive
35	Constitutive androstane receptor (CAR)	Inactive	Inactive	Inactive	Inactive	Inactive	Inactive
36	Pregnane X receptor (PXR)	Active	Active	Inactive	Active	Inactive	Inactive
37	NADH-quinone oxidoreductase (NADHOX)	Inactive	Inactive	Inactive	Inactive	Inactive	Inactive
38	Voltage gated sodium channel (VGSC)	Inactive	Inactive	Inactive	Inactive	Inactive	Inactive
39	Na+ /I- symporter (NIS)	Inactive	Inactive	Inactive	Inactive	Inactive	Inactive

**No**	**Target**	**MS7**	**MS8**	**MS9**	**MS10**	**MS11**
1	Hepatotoxicity	Inactive	Inactive	Inactive	Inactive	Inactive
2	Neurotoxicity	Inactive	Inactive	Active	Inactive	Inactive
3	Nephrotoxicity	Active	Active	Active	Active	Active
4	Respiratory toxicity	Active	Active	Active	Active	Active
5	Cardiotoxicity	Inactive	Inactive	Inactive	Inactive	Inactive
6	Carcinogenicity	Active	Active	Inactive	Active	Active
7	Immunotoxicity	Inactive	Inactive	Inactive	Inactive	Inactive
8	Mutagenicity	Inactive	Inactive	Inactive	Inactive	Inactive
9	Cytotoxicity	Inactive	Inactive	Inactive	Inactive	Inactive
10	BBB-barrier	Active	Active	Active	Active	Active
11	Ecotoxicity	Inactive	Inactive	Active	Active	Active
12	Clinical toxicity	Active	Active	Active	Active	Active
13	Nutritional toxicity	Active	Active	Inactive	Inactive	Active
14	Aryl hydrocarbon Receptor (AhR)	Inactive	Inactive	Inactive	Inactive	Active
15	Androgen Receptor (AR)	Inactive	Inactive	Inactive	Inactive	Inactive
16	Androgen Receptor Ligand Binding Domain (AR-LBD)	Inactive	Inactive	Inactive	Inactive	Inactive
17	Aromatase	Inactive	Inactive	Inactive	Inactive	Inactive
18	Estrogen Receptor Alpha (ER)	Inactive	Inactive	Inactive	Inactive	Inactive
19	Estrogen Receptor Ligand Binding Domain (ER-LBD)	Inactive	Inactive	Inactive	Inactive	Inactive
20	Peroxisome Proliferator Activated Receptor Gamma (PPAR-Gamma)	Inactive	Inactive	Inactive	Inactive	Inactive
21	Nuclear factor (erythroid-derived 2)-like 2/antioxidant responsive element (nrf2/ARE)	Inactive	Inactive	Inactive	Inactive	Inactive
22	Heat shock factor response element (HSE)	Inactive	Inactive	Inactive	Inactive	Inactive
23	Mitochondrial Membrane Potential (MMP)	Inactive	Inactive	Inactive	Inactive	Inactive
24	Phosphoprotein (Tumor Supressor) p53	Inactive	Inactive	Inactive	Inactive	Inactive
25	ATPase family AAA domain-containing protein 5 (ATAD5)	Inactive	Inactive	Inactive	Inactive	Inactive
26	Thyroid hormone receptor alpha (THRα)	Inactive	Inactive	Inactive	Inactive	Inactive
27	Thyroid hormone receptor beta (THRβ)	Inactive	Inactive	Inactive	Inactive	Inactive
28	Transtyretrin (TTR)	Inactive	Inactive	Inactive	Inactive	Inactive
29	Ryanodine receptor (RYR)	Inactive	Inactive	Inactive	Inactive	Inactive
30	GABA receptor (GABAR)	Inactive	Inactive	Inactive	Inactive	Inactive
31	Glutamate N-methyl-D-aspartate receptor (NMDAR)	Inactive	Inactive	Inactive	Inactive	Inactive
32	alpha-amino-3-hydroxy-5-methyl-4-isoxazolepropionate receptor (AMPAR)	Inactive	Inactive	Inactive	Inactive	Inactive
33	Kainate receptor (KAR)	Inactive	Inactive	Inactive	Inactive	Inactive
34	Achetylcholinesterase (AChE)	Active	Inactive	Active	Inactive	Active
35	Constitutive androstane receptor (CAR)	Inactive	Inactive	Inactive	Inactive	Inactive
36	Pregnane X receptor (PXR)	Inactive	Inactive	Inactive	Inactive	Inactive
37	NADH-quinone oxidoreductase (NADHOX)	Inactive	Inactive	Inactive	Inactive	Inactive
38	Voltage gated sodium channel (VGSC)	Inactive	Inactive	Inactive	Inactive	Inactive
39	Na+ /I- symporter (NIS)	Inactive	Inactive	Inactive	Inactive	Inactive

The extended toxicity endpoint panel further provides mechanistic clues regarding potential neuropharmacological and metabolic interaction liabilities for the selected compounds. GABA receptor activity was predicted as active only for MS2, which may indicate a unique potential for CNS-related modulation compared to other compounds. Acetylcholinesterase (AChE) activity was predicted as active for MS1, MS3, MS4, and MS5, and also active for MS7, MS9, and MS11, suggesting that cholinergic pathway modulation may represent a recurring feature among several Mitragyna speciosa metabolites. Such AChE-related predictions may be relevant to neurological signaling, cognitive modulation, or off-target CNS effects depending on exposure and dose. Pregnane X receptor (PXR) activity was predicted as active for MS1, MS2, and MS4, indicating possible involvement in xenobiotic metabolism regulation and potential drug–drug interaction risk. The absence of CAR activity across all compounds suggests that nuclear receptor-mediated induction patterns may be more specific to PXR rather than broader hepatic enzyme regulation. For the second compound set, neurotoxicity was predicted as active only for MS9, while MS7, MS8, MS10, and MS11 were inactive, indicating heterogeneity in CNS toxicity risk across the broader panel. Nephrotoxicity was predicted as active for all compounds in the MS7–MS11 group, suggesting a consistent kidney-related safety concern that should be prioritized for validation. Respiratory toxicity, BBB-barrier permeability, and clinical toxicity were also predicted as active for all compounds in the MS7–MS11 group, reinforcing the need for cautious progression of these candidates. Carcinogenicity was predicted as active for MS7, MS8, MS10, and MS11, while MS9 was inactive, indicating that carcinogenicity risk may depend on subtle structural differences. AhR activity was predicted as active only for MS11, which may indicate a unique potential for xenobiotic response pathway activation among the second group. Ecotoxicity was predicted as active for MS9, MS10, and MS11, suggesting that later-stage development should consider environmental safety if scale-up or disposal becomes relevant. The consistent inactivity of endocrine receptor targets and genotoxicity-related endpoints across both compound sets supports a relatively low predicted risk for hormone disruption and DNA-damage signaling. However, the repeated presence of respiratory toxicity and clinical toxicity alerts across almost all compounds indicates that safety cannot be inferred solely from the absence of mutagenicity or cytotoxicity flags. These in silico results should therefore be treated as a screening-level risk map rather than definitive toxicity conclusions. Follow-up experimental evaluation such as acute toxicity studies, kidney and lung function markers, and behavioral observation would be required to confirm the predicted liabilities. The toxicity profile can also be used to prioritize safer candidates by selecting compounds with fewer active endpoints while maintaining predicted efficacy against diabetes-related targets.

### 3.8 Molecular Docking Preparation and Validation

The compound–target selection for molecular docking was guided by the network pharmacology outputs, which prioritized key metabolites from *Mitragyna speciosa* associated with high-ranking hub proteins. Three representative compounds were selected, namely mitraphylline (MS1), corynoxine (MS7), and corynantheidine (MS9), based on their predicted interactions with multiple hub targets. Mitraphylline exhibited the broadest target spectrum, being associated with EGFR, PIK3CA, JAK2, SRC, and ERBB2, indicating its potential to modulate interconnected signaling pathways relevant to diabetes-related molecular mechanisms. Corynoxine was predicted to interact with EGFR, JAK2, and PIK3CA, suggesting a more focused but still biologically meaningful target profile involving receptor tyrosine kinase and inflammatory signaling. Corynantheidine was associated with PIK3CA, ERBB2, and JAK2, supporting its potential role in regulating PI3K-mediated metabolic pathways and kinase-driven signal transduction. The selection strategy emphasized compounds capable of influencing multiple central nodes rather than single targets, which aligns with the multitarget nature of phytochemical-based therapeutic interventions. Such a systems-level approach enhances the relevance of docking analyses by ensuring that evaluated ligand–protein interactions reflect biologically meaningful regulatory hubs. The predicted hub targets linked to each selected compound are presented in Table 6, highlighting that MS1 showed the most extensive target coverage compared to MS7 and MS9. In addition, the protein receptors selected for docking included EGFR (PDB ID: 4HJO), PIK3CA (6PYS), JAK2 (3FUP), SRC (7WF5), and ERBB2 (3PP0), all derived from Homo sapiens and resolved using X-ray diffraction methods. Structural details such as native ligands, experimental method, and crystallographic resolution are provided in Table 7, supporting the reliability of these receptors for structure-based simulations. The crystallographic resolutions, ranging from 1.80 Å to 2.75 Å, indicate sufficient structural quality for accurate docking pose prediction. High-resolution structures, such as SRC with a resolution of 1.80 Å, provide precise atomic coordinates that improve the identification of key binding interactions. The presence of native ligands in each crystal structure further supports accurate binding site identification and enables validation of the docking protocol. These receptors represent critical nodes in growth factor signaling, PI3K pathway regulation, and inflammatory kinase cascades, which are strongly implicated in the pathophysiology of diabetes mellitus. Therefore, the compound–target pairing and receptor selection establish a mechanistically grounded framework for subsequent docking validation and interaction analysis.

**Table 6:** Selected Compounds and Their Predicted Hub Targets for Molecular Docking Analysis.

**No**	**Compound Code**	**Selected Compound**	**Selected Target Prediction**
1	MS1	Mitraphylline	EGFR, PIK3CA, JAK2, SRC, ERBB2
2	MS7	Corynoxine	EGFR, JAK2, PIK3CA
3	MS9	Corynantheidine	PIK3CA, ERBB2, JAK2

**Table 7:** Selected Target Proteins and Their Crystal Structures Used for Molecular Docking.

**No**	**Target Receptor**	**PDB ID**	**Native Ligand**	**Organism(s)**	**Method**	**Resolution**	**Reference**
1	EGFR	4HJO	[6,7-bis(2-methoxy-ethoxy)quinazoline-4-yl]-(3-ethynylphenyl)amine	Homo sapiens	X-Ray Diffraction	2.75 Å	Ochoa, Rojas [32]
2	PIK3CA	6PYS	(3S)-3-benzyl-3-methyl-5-[5-(2-methylpyrimidin-5-yl)pyrazolo[1,5-*a*]pyrimidin-3-yl]-1,3-dihydro-2H-indol-2-one	Homo sapiens	X-Ray Diffraction	2.19 Å	Roney, Aluwi [40]
3	JAK2	3FUP	3-{(3 R,4 R)-4-methyl-3-[methyl(7H-pyrrolo[2,3-*d*]pyrimidin-4-yl)amino]piperidin-1-yl}-3-oxopropanenitrile	Homo sapiens	X-Ray Diffraction	2.40 Å	Lv et al., [28]
4	SRC	7WF5	3-(imidazo[1,2-*b*]pyridazin-3-ylethynyl)-4-methyl-N-{4-[(4-methylpiperazin-1-yl)methyl]-3-(trifluoromethyl)phenyl}benzam ide	Homo sapiens	X-Ray Diffraction	1.80 Å	Thapa et al., [51]
5	ERBB2	3PP0	2-{2-[4-({5-chloro-6-[3-(trifluoromethyl)phenoxy]pyridin-3-yl}amino)-5H-pyrrolo[3,2-*d*]pyrimidin-5-yl]ethoxy}ethanol	Homo sapiens	X-Ray Diffraction	2.25 Å	Prakash et al., [37]

Docking parameter configuration and protocol validation were established through grid box definition and re-docking evaluation to ensure reproducibility and methodological accuracy. A consistent grid spacing of 0.375 Å was applied across all target proteins, ensuring uniform sampling resolution during docking simulations. The grid center dimensions were maintained at 64 Å × 60 Å × 60 Å, providing adequate coverage of the active site regions for each receptor and enabling comparable docking conditions among targets. Differences in grid box coordinates reflected variations in binding pocket locations among the selected proteins, ensuring that each docking search space was appropriately positioned around the native ligand binding region. The detailed grid box settings and validation outcomes for each receptor are shown in Table 8, which provides the RMSD values obtained from re-docking analysis. Re-docking RMSD values were used as a key indicator of docking reliability by assessing the similarity between predicted ligand poses and their corresponding crystallographic conformations. The RMSD values ranged from 0.61 Å to 1.66 Å, demonstrating acceptable reproduction of native binding modes and confirming the suitability of the docking workflow. PIK3CA showed the lowest RMSD value (0.61 Å), indicating excellent agreement between predicted and experimental ligand poses and strong confidence in docking accuracy for this target. SRC also displayed a low RMSD (0.77 Å), supporting robust docking performance, particularly for high-resolution structures. EGFR exhibited an RMSD of 1.08 Å, which remains within the commonly accepted threshold for docking validation studies. ERBB2 produced an RMSD value of 1.31 Å, suggesting reliable binding site definition and consistent pose prediction. JAK2 showed the highest RMSD (1.66 Å), which may reflect higher flexibility of the binding environment but still indicates acceptable re-docking performance. RMSD values below 2.0 Å are generally considered evidence of a valid docking protocol, supporting the reliability of subsequent ligand screening. These validation results confirm that the selected docking parameters and grid configurations are appropriate for evaluating binding affinity and interaction profiles of *Mitragyna speciosa* compounds against the identified hub targets.

**Table 8:** Molecular Docking Parameters and Validation Results for Selected Target Proteins.

**No**	**Target Receptor**	**Grid Box**	**Spacing**	**Grid Center**	**RMSD**
1	EGFR	24.407 Å × 9.151 Å × -0.636 Å	0.375 Å	64 Å × 60 Å × 60 Å	1.08 Å
2	PIK3CA	-19.177 Å × 11.456 Å × 28.025 Å	0.375 Å	64 Å × 60 Å × 60 Å	0.61 Å
3	JAK2	-58.933 Å × 7.865 Å × 0.58 Å	0.375 Å	64 Å × 60 Å × 60 Å	1.66 Å
4	SRC	0.357 Å × 33.497 Å × -7.134 Å	0.375 Å	64 Å × 60 Å × 60 Å	0.77 Å
5	ERBB2	16.387 Å × 17.394 Å × 26.218 Å	0.375 Å	64 Å × 60 Å × 60 Å	1.31 Å

### 3.9 Molecular Docking Analysis of Selected Compounds

The molecular docking evaluation revealed distinct binding preferences of the selected *Mitragyna speciosa* compounds across the five hub targets, as reflected by the estimated free energy of binding (ΔG) and predicted inhibition constant (Ki). Three compounds were assessed, namely mitraphylline, corynoxine, and corynantheidine, and their binding performance varied depending on the receptor context. In EGFR, corynantheidine exhibited the strongest predicted interaction among the tested ligands, with ΔG of –9.01 kcal/mol and Ki of 250.21 nM, indicating favorable binding stabilization within the kinase pocket. This binding strength exceeded the native ligand in EGFR, which showed ΔG of –7.59 kcal/mol and Ki of 2.72 µM, suggesting that corynantheidine may possess a more compatible fit for EGFR recognition. For PIK3CA, the native ligand remained the strongest binder (ΔG –10.56 kcal/mol, Ki 18.24 nM), while corynantheidine still ranked as the best among the three selected compounds (ΔG –8.24 kcal/mol, Ki 904.36 nM). Mitraphylline and corynoxine displayed weaker binding against PIK3CA, with micromolar-range Ki values, implying reduced interaction strength compared to the crystallographic inhibitor. These findings indicate that corynantheidine is a promising candidate for receptor tyrosine kinase–related mechanisms, particularly through EGFR and PI3K pathway modulation. The binding affinity trends and predicted Ki values for each target are detailed in Table 9, supporting compound prioritization based on quantitative docking metrics.

**Table 9:** Molecular Docking Results of Selected *Mitragyna speciosa* Compounds Against Hub Target Proteins.

**No**	**Target Receptor**	**Selected Compound**	**Estimated Free Energy of Binding**	**Estimated Inhibition Constant**
1	EGFR	Native Ligand	-7.59 kcal/mol	2.72 μM (micromolar)
		Mitraphylline	-7.20 kcal/mol	5.30 μM (micromolar)
		Corynoxine	-6.73 kcal/mol	11.58 μM (micromolar)
		Corynantheidine	-9.01 kcal/mol	250.21 nM (nanomolar)
2	PIK3CA	Native Ligand	-10.56 kcal/mol	18.24 nM (nanomolar)
		Mitraphylline	-7.86 kcal/mol	1.74 μM (micromolar)
		Corynoxine	-6.85 kcal/mol	9.51 μM (micromolar)
		Corynantheidine	-8.24 kcal/mol	904.36 nM (nanomolar)
3	JAK2	Native Ligand	-8.57 kcal/mol	521.36 nM (nanomolar)
		Mitraphylline	-8.77 kcal/mol	374.56 nM (nanomolar)
		Corynoxine	-7.97 kcal/mol	1.44 μM (micromolar)
		Corynantheidine	-8.56 kcal/mol	528.62 nM (nanomolar)
4	SRC	Native Ligand	-14.31 kcal/mol	32.24 pM (picomolar)
		Mitraphylline	-8.72 kcal/mol	408.23 nM (nanomolar)
		Corynoxine	-8.76 kcal/mol	381.92 nM (nanomolar)
		Corynantheidine	-8.17 kcal/mol	1.03 μM (micromolar)
5	ERBB2	Native Ligand	-10.53 kcal/mol	19.17 nM (nanomolar)
		Mitraphylline	-8.80 kcal/mol	355.20 nM (nanomolar)
		Corynoxine	-6.54 kcal/mol	16.12 μM (micromolar)
		Corynantheidine	-8.64 kcal/mol	462.32 nM (nanomolar)

A different binding pattern was observed for JAK2, SRC, and ERBB2, where the selected alkaloids showed moderate affinity but did not consistently surpass the native ligands. In JAK2, mitraphylline demonstrated the strongest predicted binding among the tested compounds, with ΔG of –8.77 kcal/mol and Ki of 374.56 nM, slightly improving upon the native ligand (ΔG –8.57 kcal/mol, Ki 521.36 nM). This result suggests that mitraphylline may be particularly relevant for kinase signaling regulation through JAK2 inhibition, which is closely associated with inflammatory and metabolic dysregulation in diabetes. In SRC, the native ligand exhibited exceptionally strong binding (ΔG –14.31 kcal/mol, Ki 32.24 pM), while all three test compounds showed weaker nanomolar-to-micromolar affinities, indicating limited competitiveness in occupying the SRC binding pocket. For ERBB2, the native ligand remained the strongest reference (ΔG –10.53 kcal/mol, Ki 19.17 nM), whereas mitraphylline and corynantheidine produced moderate binding profiles with Ki values of 355.20 nM and 462.32 nM, respectively. Corynoxine consistently showed the weakest affinity across several targets, particularly ERBB2 with Ki reaching 16.12 µM, suggesting less favorable pocket accommodation and fewer stabilizing interactions. These results imply that the selected *Mitragyna speciosa* metabolites may not function as universal inhibitors for all hub targets, but rather exhibit selective preferences depending on receptor architecture. Such target-dependent selectivity is important because it supports a multitarget but non-uniform pharmacological profile that may be beneficial for complex metabolic diseases. Therefore, mitraphylline may be prioritized for JAK2-centered mechanisms, whereas corynantheidine may be prioritized for EGFR- and PI3K-associated signaling modulation.

Residue-level interaction mapping provides a detailed structural rationale for the observed docking affinities by delineating the specific amino acid contacts stabilizing each ligand within the kinase domains of the hub targets (Table 10 and Table 11). For EGFR, both the native ligand and plant-derived alkaloids consistently engaged canonical ATP-binding site residues, including ALA719, LYS721, VAL702, LEU820, and MET769, confirming that all compounds occupied the conserved kinase cleft. Corynantheidine exhibited an expanded interaction profile, forming additional hydrogen bonds with THR766 and THR830 and hydrophobic contacts with MET742, LEU753, and LEU764, which likely enhanced anchoring strength and explains its more favorable ΔG and Ki values. In PIK3CA, recurrent involvement of TRP780, ILE800, VAL851, MET922, and ILE932 across ligands reflected stable accommodation within the PI3K catalytic pocket. Corynantheidine formed multiple hydrogen bonds with ARG770, SER854, VAL851, and GLN859, accompanied by extensive hydrophobic interactions, suggesting stronger pocket engagement compared with the native ligand and corynoxine. The JAK2 interaction landscape was dominated by hydrophobic residues such as LEU855, LEU983, VAL863, and ALA880, consistent with typical kinase inhibitor binding, while mitraphylline and corynantheidine uniquely interacted with ASP994 through hydrogen bonding or electrostatic contacts, providing additional polar stabilization. For SRC, binding involved conserved residues including LYS295, HIS384, MET314, LEU322, and ASP404, where the native ligand displayed a broader hydrogen-bonding network than the plant alkaloids, consistent with its substantially lower Ki value. In ERBB2, residues such as MET801, CYS805, LYS753, LEU852, VAL734, and THR862 confirmed docking within the HER2 kinase pocket, although the native ligand again demonstrated a denser and more diverse interaction footprint. The recurrence of conserved kinase-site residues across all targets supports a shared ATP-pocket binding mechanism, while variations in hydrogen bonding patterns and contact diversity account for differences in predicted binding strength among the ligands.

**Table 10:** Predicted Protein–Ligand Interaction Residues of Selected Compounds with Hub Target Proteins.

**No**	**Target Receptor**	**Native Ligand**			**Mitraphylline**		
		**Residue**	**Category**	**2D Orientation**	**Residue**	**Category**	**2D Orientation**
1	EGFR	A:MET769:N A:GLN767:O A:LEU694:O A:MET769:O A:PRO770:O A:MET769:O A:PRO770:O A:LEU694:CD1 A:LEU820:CD1 A:ALA719 A:LYS721 A:LEU764 A:VAL702 A:ALA719 A:LYS721 A:ALA719 A:MET769 A:LEU820	Hydrogen Bond Hydrogen Bond Hydrogen Bond Hydrogen Bond Hydrogen Bond Hydrogen Bond Hydrogen Bond Hydrophobic Hydrophobic Hydrophobic Hydrophobic Hydrophobic Hydrophobic Hydrophobic Hydrophobic Hydrophobic Hydrophobic Hydrophobic		A:THR830:OG1 A:ASP831:OD2 A:ASP831:OD1 A:VAL702 A:ALA719 A:LYS721 A:LEU820 A:LEU834 A:VAL702 A:LYS721 A:LEU820 A:LEU753	Hydrogen Bond Hydrogen Bond Hydrogen Bond Hydrophobic Hydrophobic Hydrophobic Hydrophobic Hydrophobic Hydrophobic Hydrophobic Hydrophobic Hydrophobic	
2	PIK3CA	A:LYS802:NZ A:VAL851:O A:ILE848:CG2 A:TRP780 A:TYR836 A:ILE932 A:ILE800 A:ILE932 A:VAL850 A:VAL851 A:ILE932 A:VAL850 A:VAL851 A:MET922 A:MET772 A:PRO778 A:ILE800	Hydrogen Bond Hydrogen Bond Hydrophobic Hydrophobic Hydrophobic Hydrophobic Hydrophobic Hydrophobic Hydrophobic Hydrophobic Hydrophobic Hydrophobic Hydrophobic Hydrophobic Hydrophobic Hydrophobic Hydrophobic		A:VAL851:N A:SER854:OG A:THR856:N A:SER854:O A:GLU849:O A:MET922 A:ILE932 A:MET922 A:ILE800 A:TRP780	Hydrogen Bond Hydrogen Bond Hydrogen Bond Hydrogen Bond Hydrogen Bond Hydrophobic Hydrophobic Hydrophobic Hydrophobic Hydrophobic	
3	JAK2	A:LEU932:N A:ARG980:NE A:ARG980:O A:ASN981:OD1 A:LEU855:O A:LEU932:O A:LEU855:CD1 A:LEU983:CD1 A:LEU983:CD2 A:VAL863 A:LEU983 A:VAL863 A:ALA880 A:MET929 A:ALA880	Hydrogen Bond Hydrogen Bond Hydrogen Bond Hydrogen Bond Hydrogen Bond Hydrogen Bond Hydrophobic Hydrophobic Hydrophobic Hydrophobic Hydrophobic Hydrophobic Hydrophobic Hydrophobic Hydrophobic		A:LEU932:N A:SER936:N A:ARG980:O A:LEU855:O A:ASP994:OD1 A:LEU855 A:VAL863 A:ALA880 A:ALA880 A:ALA880 A:LEU983 A:LEU983 A:LEU855 A:TYR931 A:VAL863	Hydrogen Bond Hydrogen Bond Hydrogen Bond Hydrogen Bond Electrostatic Hydrophobic Hydrophobic Hydrophobic Hydrophobic Hydrophobic Hydrophobic Hydrophobic Hydrophobic Hydrophobic Hydrophobic	
4	SRC	A:ASP404:N A:GLU339:O A:HIS384:O A:HIS384:O A:LYS295:NZ A:LEU273:CD1 A:THR338:CG2 A:LEU393:CD1 A:LEU393:CD2 A:ALA293 A:LYS295 A:LEU322 A:VAL402 A:HIS384 A:LYS295 A:VAL323 A:ALA293 A:ALA293 A:MET314	Hydrogen Bond Hydrogen Bond Hydrogen Bond Hydrogen Bond Electrostatic Hydrophobic Hydrophobic Hydrophobic Hydrophobic Hydrophobic Hydrophobic Hydrophobic Hydrophobic Hydrophobic Hydrophobic Hydrophobic Hydrophobic Hydrophobic Hydrophobic		A:ASP404:N A:HIS384:O A:ASP404:OD2 A:GLU310:OE2 A:MET314 A:LEU322 A:VAL377 A:ALA403 A:LEU317 A:LEU317 A:LEU322 A:VAL313	Hydrogen Bond Hydrogen Bond Hydrogen Bond Hydrogen Bond Hydrophobic Hydrophobic Hydrophobic Hydrophobic Hydrophobic Hydrophobic Hydrophobic Hydrophobic	
5	ERBB2	A:MET801:N A:CYS805:N A:GLN799:O A:THR862:OG1 A:LEU726:CD1 A:LEU852:CD1 A:LEU852:CD2 A:PHE864 A:LYS753 A:LEU796 A:MET774 A:LEU785 A:LEU796 A:VAL734 A:ALA751 A:LYS753 A:ALA751 A:MET801 A:ALA751 A:MET774 A:LEU785 A:LEU796	Hydrogen Bond Hydrogen Bond Hydrogen Bond Hydrogen Bond Hydrophobic Hydrophobic Hydrophobic Hydrophobic Hydrophobic Hydrophobic Hydrophobic Hydrophobic Hydrophobic Hydrophobic Hydrophobic Hydrophobic Hydrophobic Hydrophobic Hydrophobic Hydrophobic Hydrophobic Hydrophobic		A:LYS753:N A:MET801:N A:THR862:OG1 A:ASP863:OD2 A:THR798:OG1 A:VAL734 A:VAL734 A:ALA751 A:ALA751 A:LYS753 A:LEU852 A:LEU852 A:LEU785	Hydrogen Bond Hydrogen Bond Hydrogen Bond Hydrogen Bond Hydrogen Bond Hydrophobic Hydrophobic Hydrophobic Hydrophobic Hydrophobic Hydrophobic Hydrophobic Hydrophobic

**Table 11:** Predicted Protein–Ligand Interaction Residues of Selected Compounds with Hub Target Proteins.

**No**	**Target Receptor**	**Corynoxine**			**Corynantheidine**		
		**Residue**	**Category**	**2D Orientation**	**Residue**	**Category**	**2D Orientation**
1	EGFR	A:LYS721:NZ A:ARG817:O A:LYS721:NZ A:VAL702:CG2 A:VAL702 A:ARG817 A:LEU820 A:VAL702	Hydrogen Bond Hydrogen Bond Hydrogen Bond;Electrostatic Hydrophobic Hydrophobic Hydrophobic Hydrophobic Hydrophobic		A:THR766:OG1 A:THR830:OG1 A:THR766:CG2 A:VAL702 A:ALA719 A:ALA719 A:LYS721 A:LYS721 A:LEU764 A:LEU834 A:LYS721 A:CYS751 A:MET742 A:LEU753	Hydrogen Bond Hydrogen Bond Hydrophobic Hydrophobic Hydrophobic Hydrophobic Hydrophobic Hydrophobic Hydrophobic Hydrophobic Hydrophobic Hydrophobic Hydrophobic Hydrophobic	
2	PIK3CA	A:TYR836:OH A:SER919:O A:ASP810:OD1 A:ILE800 A:ILE848 A:ILE932 A:PRO778 A:ILE800 A:LYS802 A:ILE848	Hydrogen Bond Hydrogen Bond Hydrogen Bond Hydrophobic Hydrophobic Hydrophobic Hydrophobic Hydrophobic Hydrophobic Hydrophobic		A:ARG770:NH2 A:SER854:N A:VAL851:O A:GLN859:OE1 A:VAL851:O A:TRP780 A:MET922 A:TRP780 A:TRP780 A:VAL850 A:VAL851 A:MET922 A:ILE800 A:VAL850	Hydrogen Bond Hydrogen Bond Hydrogen Bond Hydrogen Bond Hydrogen Bond Hydrophobic Hydrophobic Hydrophobic Hydrophobic Hydrophobic Hydrophobic Hydrophobic Hydrophobic Hydrophobic	
3	JAK2	A:ILE982:O A:GLY856:CA A:GLY858:CA A:GLY993:CA A:ASP994:OD1 A:ASP994:OD1 A:VAL863 A:VAL863 A:LEU855 A:VAL863 A:LEU983	Hydrogen Bond Hydrogen Bond Hydrogen Bond Hydrogen Bond Hydrogen Bond Hydrogen Bond Hydrophobic Hydrophobic Hydrophobic Hydrophobic Hydrophobic		A:GLY856:CA A:GLY858:CA A:ASP994:OD1 A:GLY993:O A:LYS857:O A:LEU855:CD1 A:LEU983:CD1 A:LEU983:CD1 A:VAL863 A:VAL863 A:LEU983 A:VAL863 A:LEU855 A:VAL863	Hydrogen Bond Hydrogen Bond Hydrogen Bond Hydrogen Bond Hydrogen Bond Hydrophobic Hydrophobic Hydrophobic Hydrophobic Hydrophobic Hydrophobic Hydrophobic Hydrophobic Hydrophobic	
4	SRC	A:LEU317:O A:LEU317 A:LEU322 A:VAL377 A:VAL402 A:VAL402 A:LEU322 A:HIS384 A:HIS384	Hydrogen Bond Hydrophobic Hydrophobic Hydrophobic Hydrophobic Hydrophobic Hydrophobic Hydrophobic Hydrophobic		A:ALA390:O A:GLN275:O A:LEU393:CD1 A:LEU393:CD2 A:LEU273 A:LEU273 A:VAL281 A:VAL281 A:LEU393 A:PHE405 A:LEU273 A:VAL281 A:ALA293 A:ALA293	Hydrogen Bond Hydrogen Bond Hydrophobic Hydrophobic Hydrophobic Hydrophobic Hydrophobic Hydrophobic Hydrophobic Hydrophobic Hydrophobic Hydrophobic Hydrophobic Hydrophobic	
5	ERBB2	A:SER728:N A:ASP808:OD1 A:ASN850:OD1 A:LEU852:CD2 A:CYS805 A:CYS805 A:LEU807 A:ARG849 A:VAL734 A:CYS805	Hydrogen Bond Hydrogen Bond Hydrogen Bond Hydrophobic Hydrophobic Hydrophobic Hydrophobic Hydrophobic Hydrophobic Hydrophobic		A:GLN799:O A:THR862:OG1 A:VAL734 A:VAL734 A:ALA751 A:LYS753 A:LEU852 A:LEU726 A:VAL734 A:VAL734 A:LYS753 A:LYS753	Hydrogen Bond Hydrogen Bond Hydrophobic Hydrophobic Hydrophobic Hydrophobic Hydrophobic Hydrophobic Hydrophobic Hydrophobic Hydrophobic Hydrophobic	

### 3.10 In Vitro Enzyme Inhibition Analysis

The in vitro enzyme inhibition results presented in Table 12 demonstrate a distinct difference in inhibitory potency of the kratom extract toward α-glucosidase and α-amylase. The kratom extract exhibited moderate inhibitory activity against alpha-glucosidase with an IC₅₀ value of 48.49 ppm, indicating a measurable capacity to suppress carbohydrate hydrolysis at the intestinal level. This activity, although lower than the standard drug acarbose, which showed an IC₅₀ of 0.01 ppm, still reflects the presence of bioactive constituents capable of interacting with the enzyme active site. The relatively higher IC₅₀ value suggests that a higher concentration of extract is required to achieve comparable inhibition, which is common for crude extracts containing a mixture of compounds. In contrast, the inhibitory activity against α-amylase was considerably weaker, as indicated by a much higher IC₅₀ value of 17,907.37 ppm. This value was even higher than that of acarbose, which exhibited an IC₅₀ of 8,644.76 ppm, suggesting limited effectiveness of the extract in inhibiting starch hydrolysis. The disparity between α-glucosidase and α-amylase inhibition indicates a degree of enzyme selectivity exhibited by the kratom extract. Selective inhibition of α-glucosidase is considered advantageous because it may reduce postprandial glucose spikes without significantly interfering with early-stage starch digestion. Excessive inhibition of α-amylase is often associated with gastrointestinal side effects such as bloating and diarrhea. Therefore, the observed selectivity toward α-glucosidase may provide a favorable pharmacological profile for managing postprandial hyperglycemia. These findings suggest that the kratom extract may act as a mild but selective modulator of carbohydrate-digesting enzymes.

**Table 12:** Inhibitory Activity (IC₅₀) of Kratom Extract Against Alpha-Glucosidase and Alpha-Amylase.

**No**	**Assay**	**Kratom Extract IC**_**50**_**(ppm)**	**Comparator**	**Comparator IC**_**50**_**(ppm)**
1	Alpha-Glucosidase	48.49	Acarbose	0.01
2	Alpha-Amylase	17,907.37	Acarbose	8,644.76

The observed in vitro inhibitory activity can be correlated with the molecular docking results discussed previously, particularly the interaction of selected alkaloids with kinase-related targets involved in metabolic regulation. Although the docking study focused on protein targets such as EGFR, PI3K, and JAK2, the presence of strong binding interactions and favorable ΔG values suggests that these compounds possess sufficient structural features to interact with other enzyme systems. The moderate α-glucosidase inhibition may be attributed to the ability of alkaloid constituents to form hydrogen bonds and hydrophobic interactions within enzyme active sites. Compounds such as corynantheidine, which demonstrated strong binding affinity in docking studies, may contribute significantly to the observed biological activity. The weaker α-amylase inhibition may indicate suboptimal interaction of these compounds with the catalytic residues responsible for starch breakdown. This difference may arise from variations in enzyme pocket architecture, where α-glucosidase provides a more compatible binding environment for the alkaloid structures. Furthermore, the complex composition of the extract may lead to synergistic or antagonistic interactions among its constituents, influencing overall inhibitory activity. The integration of docking and in vitro findings supports the notion that kratom-derived compounds exhibit multitarget but selective biological effects. This selectivity is particularly relevant in the context of diabetes management, where modulation of multiple pathways is often required. The results reinforce the potential of *Mitragyna speciosa* as a source of bioactive compounds for antidiabetic applications. Further studies involving isolation of individual compounds and mechanistic enzyme assays are necessary to validate these findings and improve potency.

## 4 Discussions

The present findings provide a coherent explanation of the antidiabetic potential of *Mitragyna speciosa* by integrating metabolite profiling, network pharmacology, and docking-based target validation. LC–MS/MS analysis demonstrated that the extract contains multiple metabolites with varying relative abundance, supporting the concept that kratom acts through a multi-compound mechanism rather than a single active principle. This chemical diversity is highly relevant because diabetes mellitus involves multiple pathological layers, including insulin resistance, oxidative stress, inflammation, and metabolic signaling imbalance [44]. A previous LC/ESI/TOF-MS study reported that *Mitragyna speciosa* extract is abundantly rich in polyphenolic constituents, particularly flavonoid and phenolic glycosides, suggesting that non-alkaloid metabolites may also contribute to biological effects beyond the well-known indole alkaloids [11]. Such phytochemical complexity supports the rationale for applying a network-based approach, since different compound groups may influence different targets within the diabetes-associated biological system. The observed dominance of major alkaloids such as mitragynine and mitrafoline in the current dataset further suggests that indole alkaloids remain key contributors to pharmacological activity. However, minor metabolites should not be underestimated because low-abundance compounds may still exhibit strong binding toward central proteins that regulate disease progression. The presence of structurally diverse scaffolds also increases the likelihood of complementary interactions across receptors, kinases, and metabolic regulators. This is consistent with the therapeutic logic of botanical extracts, where cumulative activity may arise from parallel modulation of oxidative, inflammatory, and metabolic pathways. Therefore, LC–MS/MS results in this work serve not only as a compositional confirmation but also as a mechanistic foundation for downstream prioritization of diabetes-relevant targets. The chemical evidence supports the plausibility of multitarget intervention, which is particularly important for chronic metabolic disorders. Hence, this step provides a strong justification for interpreting subsequent network pharmacology and docking outputs as biologically meaningful.

The network pharmacology component strengthened this interpretation by showing that the predicted compound-related targets overlap with diabetes-associated targets and form a connected protein–protein interaction system. The resulting network was not randomly dispersed, indicating that the shared targets participate in coordinated signaling pathways relevant to diabetes pathogenesis. Hub gene screening identified EGFR, PIK3CA, JAK2, SRC, and ERBB2 as key nodes, implying that kinase-centered regulation may be an important mechanistic axis influenced by kratom metabolites. These hubs are biologically relevant because PI3K signaling is closely linked to insulin action and glucose uptake, while JAK-related signaling contributes to cytokine-mediated insulin resistance and metabolic inflammation. Functional enrichment further supported this pattern by emphasizing protein phosphorylation, receptor signaling, and membrane-associated molecular functions that regulate cellular responses to extracellular metabolic cues. Experimental evidence from muscle cell assays has shown that *Mitragyna speciosa* extracts and mitragynine significantly increased glucose uptake in rat L8 myotubes, and the effect was reduced by wortmannin and SB203580, indicating involvement of PI3K and p38 MAPK pathways [39]. This mechanistic observation supports the network identification of PIK3CA-related signaling as a relevant hub pathway and suggests that kratom may enhance glucose disposal in insulin-sensitive tissues. The lack of additive effect when co-incubated with insulin also implies that the extract may partially converge with insulin-dependent signaling rather than acting through an entirely independent route. Such signaling convergence is important because impaired peripheral glucose uptake is a major contributor to hyperglycemia in type 2 diabetes mellitus (T2DM). Therefore, the network results provide a logical framework for selecting hub proteins for structure-based docking, as these targets represent high-impact control points in diabetes regulation. The hub prioritization is also consistent with the concept that moderate modulation of central signaling nodes can translate into meaningful systemic outcomes. This provides a mechanistic bridge between chemical diversity and functional relevance in diabetes models. As a result, the network pharmacology outputs in this study are not only computational predictions but also align with experimentally observed glucose-uptake effects reported in the literature.

Molecular docking analysis provided structural-level support for the network-derived hub selection by demonstrating that selected kratom alkaloids can bind to kinase-associated targets with target-dependent affinity patterns. Validation of the docking protocol through acceptable re-docking RMSD values supports the reliability of predicted poses and comparative interpretation of binding scores. Corynantheidine displayed the strongest predicted affinity toward EGFR, while mitraphylline showed the most favorable binding profile toward JAK2, indicating that different alkaloid scaffolds may preferentially stabilize distinct kinase binding pockets. In contrast, native ligands remained stronger for certain targets such as SRC and PIK3CA, suggesting that kratom metabolites may function as moderate binders rather than highly optimized inhibitors. This pattern remains pharmacologically plausible in botanical systems because partial modulation across multiple hubs may still yield beneficial outcomes through pathway crosstalk and additive signaling effects. The in vitro enzyme inhibition results further complement this interpretation by demonstrating measurable α-glucosidase inhibitory activity, with an IC₅₀ value in the tens of ppm range, indicating moderate potency of the crude extract. In contrast, α-amylase inhibition was substantially weaker, reflecting limited interaction of the extract components with starch-degrading enzyme systems. This selective inhibition profile is pharmacologically advantageous because preferential suppression of α-glucosidase can reduce postprandial glucose excursions while minimizing gastrointestinal side effects associated with strong alpha-amylase inhibition. The discrepancy between the two enzyme assays also suggests that kratom metabolites may exhibit structural compatibility with glycosidase-type active sites rather than broader carbohydrate-hydrolyzing enzymes [19]. Evidence from a type 2 diabetic rat model demonstrated that *Mitragyna speciosa* ethanolic extract improved blood glucose, glucose tolerance, dyslipidemia, hepatorenal biomarkers, oxidative stress indices, and pancreatic histological alterations, supporting the biological feasibility of systemic improvement through combined mechanisms [55]. Such in vivo improvements align with the present docking results, where multiple compounds exhibited measurable binding toward central regulatory targets rather than acting on a single enzyme alone. The residue interaction mapping further supports mechanistic credibility because binding poses were located within canonical kinase pocket regions that typically govern signaling regulation. This supports the interpretation that the predicted interactions are not random surface contacts but may reflect meaningful target engagement. The integration of LC–MS/MS compositional evidence, network-derived hub selection, and docking validation therefore establishes a coherent mechanistic storyline for kratom’s antidiabetic potential. Importantly, this approach allows prioritization of corynantheidine and mitraphylline as promising candidates for deeper validation, particularly in signaling-focused assays. Further work should include enzyme inhibition studies, pathway biomarker analysis, and confirmatory cellular assays to test whether the predicted hub modulation translates into measurable downstream metabolic effects. Safety evaluation also remains essential to ensure that potential therapeutic benefits are not offset by toxicity concerns during longer-term use [20].

## 5 Conclusions

This study demonstrated that *Mitragyna speciosa* contains diverse alkaloid-related metabolites with potential antidiabetic relevance, as suggested through an integrated LC–MS/MS, network pharmacology, molecular docking, and in vitro enzyme inhibition approach. LC–MS/MS profiling identified multiple compounds with distinct retention times and relative compositions, where the dominant constituents included mitrafoline (31.54%) and mitragynine (30.59%), followed by corynoxine (10.43%), an indoloquinolizine derivative (8.41%), and isopaynantheine (6.27%), while other detected metabolites such as mitraphylline (2.58%), 6-(9′-Purine-6′,8′-diyl)-2-beta-suberosanone (4.57%), 7-hydroxymitragynine (0.52%), and corynantheidine (1.75%) were present at lower proportions but remained mechanistically important. Network pharmacology analysis revealed 28 shared targets between *Mitragyna speciosa* and diabetes mellitus, and the resulting PPI network indicated strong connectivity among these proteins. Hub target screening using MCC ranking prioritized key proteins with high centrality scores, namely EGFR, PIK3CA, JAK2, SRC, and ERBB2, suggesting that kinase-driven signaling pathways represent major regulatory nodes underlying the multitarget mechanism. Molecular docking results showed target-dependent binding patterns, where corynantheidine exhibited the strongest affinity toward EGFR and mitraphylline demonstrated favorable binding against JAK2, while native ligands remained dominant for several targets but did not invalidate the relevance of the plant-derived compounds as moderate binders. Residue interaction analysis further suggested that these ligands occupied conserved kinase pockets, supporting structurally plausible and biologically relevant interaction modes. The in vitro enzyme inhibition assays provided complementary functional evidence, where the extract exhibited moderate α-glucosidase inhibitory activity with an IC₅₀ value in the tens of ppm range, while α-amylase inhibition was substantially weaker, indicating selective enzyme targeting. This selective inhibition profile is advantageous for postprandial glucose regulation, as it may reduce glucose absorption without excessive interference with starch digestion. The integration of computational and experimental findings supports a multitarget antidiabetic mechanism involving both signaling pathway modulation and carbohydrate digestion inhibition. These results highlight corynantheidine and mitraphylline as promising candidates for further investigation, particularly in enzyme-based and cell-based validation studies. Future work should focus on isolation of active compounds, mechanistic enzyme kinetics, and in vivo evaluation to confirm therapeutic efficacy and safety.

## CRediT authorship contribution statement

**Ihsan Jaya Fathurohman:** Visualization, Validation, Software, Resources, Methodology, Data curation. **Taufik Muhammad Fakih:** Writing – review & editing, Writing – original draft, Visualization, Validation, Software, Resources, Methodology, Investigation, Formal analysis, Data curation. **Khoirunnisa Muslimawati:** Visualization, Validation, Software, Resources, Methodology, Investigation, Formal analysis, Data curation. **Jajang Japar Sodik:** Writing – original draft, Visualization, Validation, Supervision, Software, Resources, Methodology, Investigation, Formal analysis, Data curation, Conceptualization. **Nabila Hadiah Akbar:** Visualization, Validation, Software, Resources, Methodology, Investigation, Formal analysis, Data curation. **Rifky Rahmadi Khaerulihsan:** Visualization, Validation, Resources, Methodology, Data curation. **Entris Sutrisno:** Writing – review & editing, Writing – original draft, Visualization, Validation, Supervision, Software, Resources, Project administration, Methodology, Investigation, Funding acquisition, Formal analysis, Data curation, Conceptualization.

## Declaration of Competing Interest

The authors declare the following financial interests/personal relationships which may be considered as potential competing interests: Entris Sutrisno reports financial support from Universitas Bhakti Kencana. Entris Sutrisno also reports an affiliation with Universitas Bhakti Kencana. The remaining authors declare that they have no known competing financial interests or personal relationships that could have appeared to influence the work reported in this paper.

## Data Availability

The authors do not have permission to share data.
